# Acetylation changes tau interactome to degrade tau in Alzheimer’s disease animal and organoid models

**DOI:** 10.1111/acel.13081

**Published:** 2019-11-25

**Authors:** Heesun Choi, Haeng Jun Kim, Jinhee Yang, Sehyun Chae, Wonik Lee, Sunwoo Chung, Jisoo Kim, Hyunjung Choi, Hyeseung Song, Chang Kon Lee, Jae Hyun Jun, Yong Jae Lee, Kyunghyeon Lee, Semi Kim, Hye‐ri Sim, Young Il Choi, Keun Ho Ryu, Jong‐Chan Park, Dongjoon Lee, Sun‐Ho Han, Daehee Hwang, Jangbeen Kyung, Inhee Mook‐Jung

**Affiliations:** ^1^ Department of Biochemistry and Biomedical Sciences College of Medicine Seoul National University Seoul Korea; ^2^ Department of Pharmacology CKD Research Institute CKD Pharmaceutical Company Seoul Korea; ^3^ Center for Plant Aging Research Institute for Basic Science DGIST Daegu Korea; ^4^ Korea Brain Bank Korea Brain Research Institute Daegu Korea; ^5^ Interdisciplinary Graduate Program in Genetic Engineering Seoul National University Seoul Korea; ^6^ Department of Medicinal Chemistry CKD Research Institute CKD Pharmaceutical Company Seoul Korea; ^7^ Department of Nonclinical Development CKD Research Institute CKD Pharmaceutical Company Seoul Korea; ^8^ Department of New Biology DGIST Daegu Korea; ^9^ Present address: Department of Bioengineering University of Pennsylvania Philadelphia PA USA; ^10^ Present address: Department of Pharmacology CKD Research Institute CKD Pharmaceutical Company Seoul Korea

**Keywords:** Alzheimer's disease, neurodegenerative diseases, tau, tau interactome, tau post‐translational modification

## Abstract

Alzheimer's disease (AD) is an age‐related neurodegenerative disease. The most common pathological hallmarks are amyloid plaques and neurofibrillary tangles in the brain. In the brains of patients with AD, pathological tau is abnormally accumulated causing neuronal loss, synaptic dysfunction, and cognitive decline. We found a histone deacetylase 6 (HDAC6) inhibitor, CKD‐504, changed the tau interactome dramatically to degrade pathological tau not only in AD animal model (ADLP^APT^) brains containing both amyloid plaques and neurofibrillary tangles but also in AD patient‐derived brain organoids. Acetylated tau recruited chaperone proteins such as Hsp40, Hsp70, and Hsp110, and this complex bound to novel tau E3 ligases including UBE2O and RNF14. This complex degraded pathological tau through proteasomal pathway. We also identified the responsible acetylation sites on tau. These dramatic tau‐interactome changes may result in tau degradation, leading to the recovery of synaptic pathology and cognitive decline in the ADLP^APT^ mice.

## Graphical abstract

In AD, as overall level of acetylation is down‐regulated, interactions of tau with indicated chaperones and E3 ligases may be insufficient to degrade pathologic tau and hardly degraded by UPS and become aggregated. CKD‐504, an HDAC6 inhibitor, elevates interactions of tau with chaperones and E3 ligases through increasing acetylation of each protein, resulting in ubiquitination and degradation of tau by UPS. Finally, degradation of tau rescues cognitive impairment in AD model mice.

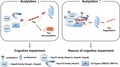

## 1 INTRODUCTION

Alzheimer's disease (AD) is the most common neurodegenerative disease that causes dementia. Intracellular accumulation of pathological tau proteins as neurofibrillary tangles (NFTs) and extracellular accumulation of beta‐amyloid (Aβ) as senile plaques are the major pathological features of AD (Querfurth & LaFerla, 2010). Physiologically, tau regulates microtubule dynamics (Qiang et al., 2018), whereas pathologically, tau is highly phosphorylated and aggregates to form NFTs in AD (Wang & Mandelkow, 2016). Hyperphosphorylated and aggregated tau leads to synapse dysfunction, mitochondrial damage, and neuronal cell death (Shafiei, Guerrero‐Munoz, & Castillo‐Carranza, 2017; Wang & Mandelkow, 2016). Accumulated tau, rather than amyloid plaques, is more correlated with cognitive decline in patients with AD (Brier et al., 2016). Several recent studies have reported that reducing endogenous tau attenuates the manifestations of Aβ toxicity, including deficits in axonal transport, seizures, cognitive impairment, and decreased survival, indicating that tau acts as an effector molecule to mediate Aβ toxicity (Roberson et al., 2007; Vossel et al., 2010). In addition, numerous clinical trials of therapeutic strategies targeting Aβ have failed (Anderson, Hadjichrysanthou, Evans, & Wong, 2017); thus, other therapies having nonamyloid mechanisms of action, especially tau‐related targets, are active areas of early‐stage drug‐development efforts (Cummings, Lee, Ritter, & Zhong, 2018). Therefore, reducing tau could be a prospective strategy for treating AD.

In AD, post‐translational modifications (PTMs) are largely changed (Marcelli et al., 2018). Acetylation is one of the important PTMs that has a variety of biological roles such as histone modulation, metabolism, and stress response. Histone deacetylase 6 (HDAC6), which is unique among histone deacetylases because of its primary cytosolic location, deacetylates several cytosolic proteins, including α‐tubulin and tau (Cook et al., 2014; Li, Shin, & Kwon, 2013). HDAC6 is increased in the brains of patients with AD (Ding, Dolan, & Johnson, 2008), suggesting that targeting HDAC6 could be a potential therapeutic strategy in AD. Indeed, reducing or inhibiting HDAC6 has been shown to attenuate cognitive deficits, decrease Aβ plaques in AβPP^swe^/PS1^ΔE9^ mice, and ameliorate tau pathologies in rTg4510 mice as well as primary cultured neurons (Cook et al., 2014; Tseng et al., 2017; Zhang et al., 2014). In addition, it is reported that acetylation of tau at several HDAC6‐regulated sites competes with phosphorylation of tau and thus inhibits its aggregation (Carlomagno et al., 2017; Cook et al., 2014). HDAC6 also plays an important role in the ubiquitin–proteasome system (UPS) and autophagy–lysosome system (ALS) (Van Helleputte, Benoy, & Van Den Bosch, 2014), both of which are known to be responsible for tau degradation (Lee, Lee, & Rubinsztein, 2013). Because tau is a substrate of HDAC6 (Cook et al., 2014), HDAC6 might regulate tau degradation. However, the specific mechanism by which HDAC6 inhibition causes tau degradation in AD is not yet known. Moreover, to our knowledge, there has been little or no investigation of the therapeutic effects of HDAC6 inhibition on human brain organoids or AD model animals exhibiting both Aβ and tau pathologies.

Here, we investigated the therapeutic effects of CKD‐504, which is a novel, selective, and highly blood–brain barrier (BBB)‐penetrating HDAC6 inhibitor. CKD‐504 effectively modified acetylation both in brain organoids formed from AD patient‐derived induced pluripotent stem cells (iPSCs) and ADLP^APT^ (Alzheimer's disease‐like pathology^APP & Tau^) mice exhibiting both Aβ and tau pathologies (Kim et al., 2018). We found that CKD‐504 reduced tau in AD patient‐iPSC‐derived brain organoids. In addition, CKD‐504 significantly reduced pathological tau and rescued synaptic pathologies and cognitive impairment in ADLP^APT^ mice. Furthermore, HDAC6 inhibition by CKD‐504 regulated the interactions of tau with chaperones and E3 ligases involved in the chaperone network, by increasing acetylation of tau, chaperones, and E3 ligases in brain organoids and in vivo, resulting in accelerated proteasomal degradation of tau. Therefore, we suggest that, by modulating acetylation of tau and a tau‐degrading chaperone network, CKD‐504 is a prominent potential therapeutic drug candidate in AD.

## 2 RESULTS

### 2.1 CKD‐504 selectively inhibits HDAC6 and is highly targeted to the brain

**Figure 1:**
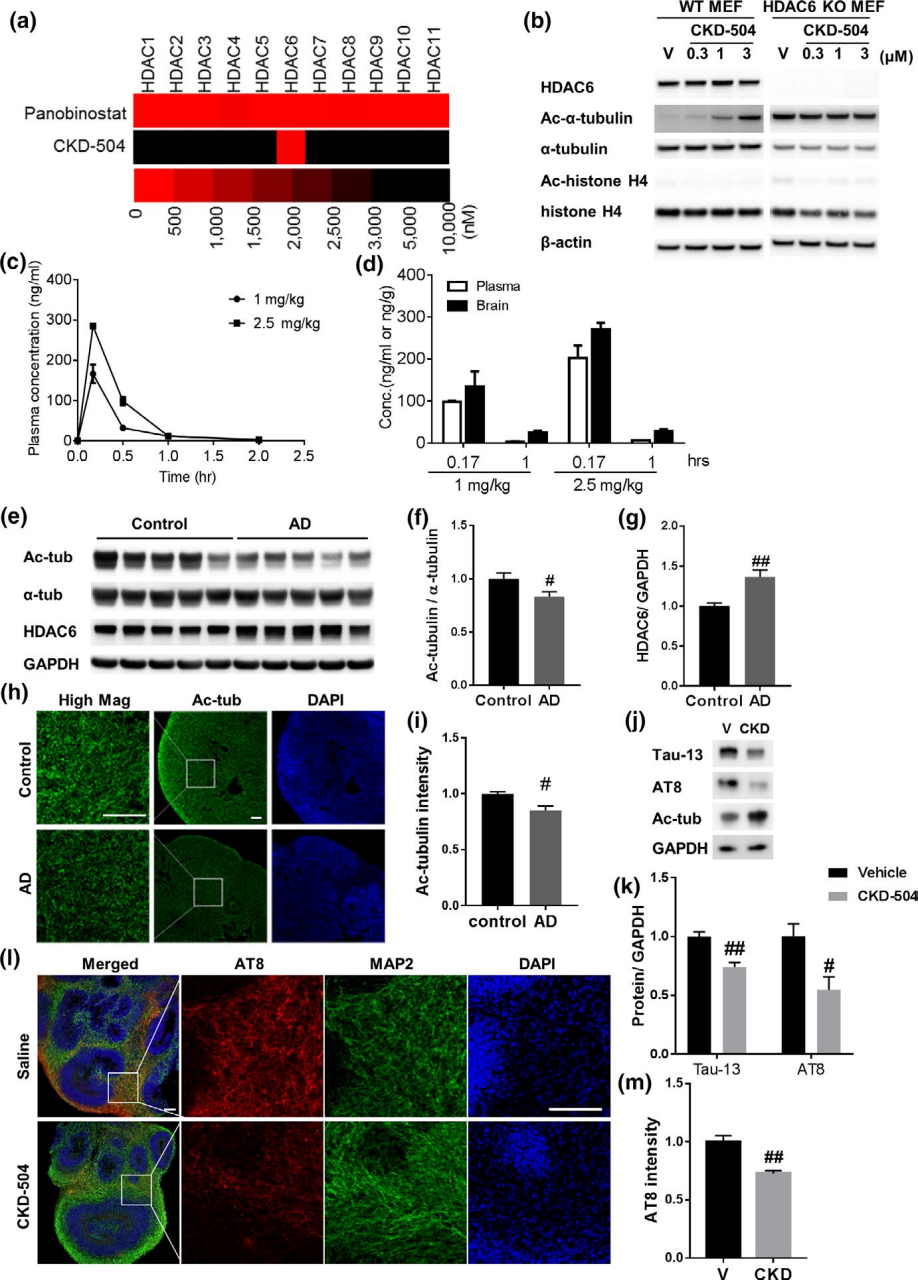
CKD‐504 selectively inhibits HDAC6 and is highly targeted to the brain, and reduces pathological tau in human brain organoids. (a) Heat maps showing in vitro IC_50_ values (nM) of CKD‐504 and panobinostat to inhibit HDAC 1–11 using recombinant human HDAC enzymes and HDAC class‐specific substrates. (b) Acetylation levels of α‐tubulin and histone H4 by treatment of CKD‐504 on WT and HDAC6 KO MEF cells. (c, d) Analysis of plasma and brain concentrations of 1 or 2.5 mg/kg of CKD‐504 after intraperitoneal (i.p.) injection to ADLP^APT^ mice (*n* = 3). Mean plasma concentrations versus time plots (c) and plasma and brain concentrations of CKD‐504 at the indicated time points following i.p. injection (d). (e‐g) The levels of acetylated α‐tubulin and HDAC6 in healthy control and AD patient‐derived human brain organoids. Representative immunoblot images (e) and quantification (f, g) (*n* = 5). (h, i) Decreased expression level of acetylated α‐tubulin in AD patient‐derived human brain organoids confirmed by immunohistochemistry. Representative images (h) and quantification (i) (*n* = 3). (j–m) Decreased amount of total and phosphorylated tau by CKD‐504 in AD patient‐derived human brain organoids. Human brain organoids were treated with 5 μM of CKD‐504 for 4 hr. Representative images (j) and quantification (k) (*n* = 5). Decreased AT8‐positive tau by immunohistochemistry. MAP2 stained for neuronal marker. Representative images (l) and quantification (m) (*n* = 4, 3). Scale bar: 100 μm. *n* means independent number of organoids. Data are expressed as mean ± *SEM*. Student's *t* tests. *#p* < .05, *##p* < .01. CKD, CKD‐504; V, vehicle

To characterize the novel HDAC6 inhibitor, CKD‐504, we first determined its selectivity for HDAC6 by measuring its half‐maximal inhibitory concentration (IC_50_) using recombinant human HDAC enzymes and HDAC class‐specific substrates. Heat maps of IC_50_ values showed that CKD‐504 selectively inhibited HDAC6 versus other HDAC isoforms compared with panobinostat, a pan‐HDAC inhibitor, which showed no selectivity among HDAC isoforms (Figure 1a). The IC_50_ value of CKD‐504 against HDAC6 was 72.2 nM. The specificity of CKD‐504 was also determined using HDAC6 knockout (KO) mouse embryonic fibroblasts (MEFs) as well as wild‐type (WT) MEFs (Jung et al., 2018) (Figure 1b). CKD‐504 increased the acetylation of α‐tubulin in a dose‐dependent manner in WT MEFs with no changes in acetyl‐histone. In HDAC6 KO MEFs, the basal levels of acetyl‐α‐tubulin were highly increased as previously reported (Zhang et al., 2008) and CKD‐504 did not affect the acetylation levels of both α‐tubulin and histone. These results further indicate that CKD‐504 selectively inhibits HDAC6. Next, we assessed pharmacokinetic parameters of CKD‐504 by measuring its concentrations in plasma and brain after a single intraperitoneal injection in ADLP^APT^ mice (Figure 1c,d, Figure S1a). Plasma and brain concentrations of CKD‐504 were increased in a dose‐dependent manner. In particular, the concentration of CKD‐504 was higher in the brain than in plasma, implying that CKD‐504 is highly targeted to the brain and shows considerable penetration of the BBB. CKD‐504 was metabolized rapidly, with a *t*_1/2_ of <0.5 hr. These data indicate that CKD‐504 is a selective HDAC6 inhibitor that is applicable to CNS diseases.

### 2.2 CKD‐504 reduces pathological tau in AD patient‐iPSC‐derived brain organoids

Because AD patient‐iPSC‐derived brain organoids expressed differentiated neuronal markers, not neural progenitor markers (data not shown) on days 65–70 after embryoid body formation, and showed similar pathological features of patients with AD (Figure 1e–i, Figure S1b,c), they are appropriate models for evaluating therapeutic effects of CKD‐504. In detail, though α‐tubulin was not altered, acetylated α‐tubulin was decreased and HDAC6 was increased in AD brain organoids compared with healthy control brain organoids, which were determined by Western blotting and immunohistochemical analyses. In addition, pathological tau detected by AT8 and AT180 antibodies was increased in AD brain organoids compared with healthy controls (data not shown). Therefore, we evaluated the therapeutic effects of CKD‐504 in AD brain organoids before testing in AD mouse models. The amounts of total and phosphorylated tau were both decreased by CKD‐504 in AD brain organoids (Figure 1j–m). These data indicate the potential of CKD‐504 to rescue tau pathologies in AD. In addition, we observed that the amount of total tau was also decreased by CKD‐504 in healthy control brain organoids (Figure S1d,e). It suggests that CKD‐504 has a great potential for reducing tau in both pathological and healthy conditions.

### 2.3 CKD‐504 rescues memory impairment and PSD‐95 levels in ADLP^APT^ mice in a preventive experimental paradigm

**Figure 2:**
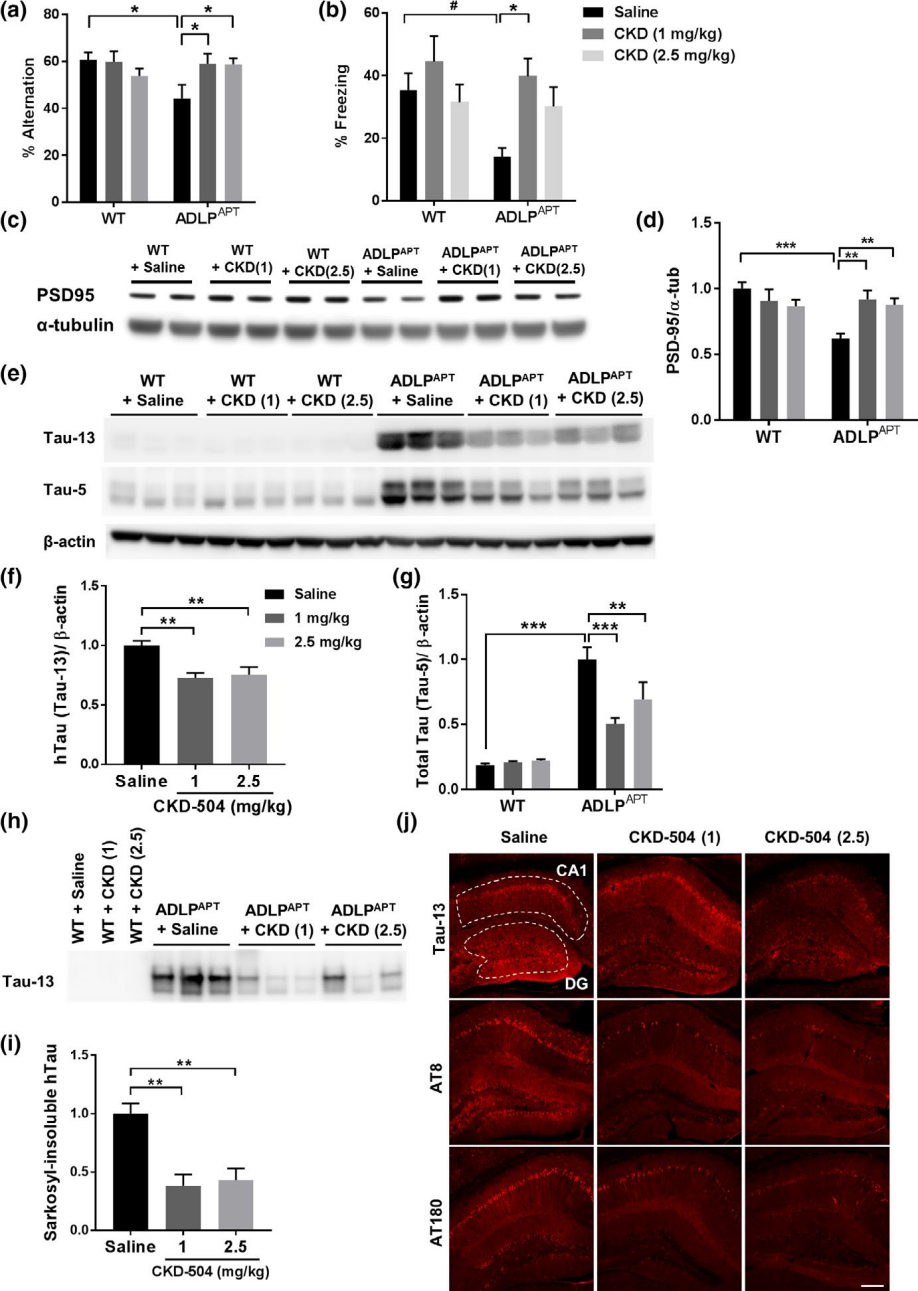
CKD‐504 recovered cognitive impairment and synaptic pathology, and reduced tau in the preventive model of ADLP^APT^ mice. (a, b) Cognitive impairment was rescued by CKD‐504 injection for 4 months in 4.5‐month‐old ADLP^APT^ mice. Quantification of Y‐maze test (a) and CFC test (b) (*n* = 7–12). (c, d) Reduced PSD‐95 levels were rescued by CKD‐504 in the cortex. Representative immunoblot images (c) and quantification (d) (*n* = 7–10). (e–i) Sarkosyl‐soluble and sarkosyl‐insoluble tau proteins were decreased by CKD‐504. Total and phosphorylated tau proteins were detected by the indicated primary antibodies. Representative immunoblot images and quantification of sarkosyl‐soluble fraction (e–g) and sarkosyl‐insoluble fraction (h, i) of hippocampal lysates from ADLP^APT^ mice. Quantification of phosphorylated tau is shown in Figure S2. (j) Total and phosphorylated tau proteins were reduced in the hippocampus as confirmed by immunohistochemistry. Representative images (j) shown here and quantifications are in Figure S2. Scale bar: 100 μm. Data are presented as means ± *SEM*. Two‐way ANOVA in (a, b, d, g) and one‐way ANOVA in (f,i) followed by Bonferroni post hoc test. *p < .05, **p < .01, ***p < .001. Student's *t* tests. *#p* < .05. CKD, CKD‐504

As an in vivo preclinical study, we tested the therapeutic efficacy of CKD‐504 in ADLP^APT^ mice. To determine the efficacy of CKD‐504 before and after memory impairment, which appears at about 6 months of age in ADLP^APT^ mice (Kim et al., 2018), we assigned two cohorts of mice to preventive or therapeutic models. In the preventive model, dose‐dependent efficacy was tested by injecting two different doses of CKD‐504 (1 and 2.5 mg/kg) for 4 months beginning at 4.5 months of age. Cognitive functions of WT and ADLP^APT^ mice were assessed using Y‐maze and contextual fear conditioning tests (Figure 2a,b). Even the low dose of CKD‐504 (1 mg/kg) rescued cognitive dysfunction in ADLP^APT^ mice. To determine the pharmacodynamics of CKD‐504 in the brain, acetylated α‐tubulin level was analyzed by Western blotting. As expected, CKD‐504 injected group showed increased levels of acetylated α‐tubulin levels, which indicates that CKD‐504 has penetrated BBB and worked well in the brain (Figure S1f,g). In addition, normal expression levels of the postsynaptic protein, PSD‐95, were retained in CKD‐504‐treated ADLP^APT^ mice (Figure 2c,d), indicating that CKD‐504 might protect synaptic proteins from damage. These data show that CKD‐504 has preventive potential in ADLP^APT^ mice.

### 2.4 CKD‐504 reduces pathological tau in ADLP^APT^ mice in a preventive experimental paradigm

Next, we sought to determine whether CKD‐504 affected Aβ or tau pathologies, which are possible mechanisms by which it might prevent memory impairment. To measure Aβ plaques, we performed immunohistochemistry using the antibody, 4G8 (Figure S1h–j). Aβ plaques were not significantly reduced in ADLP^APT^ mice by CKD‐504, although it caused a tendency toward a decrease in Aβ plaques in the dentate gyrus. Neuroinflammation, assessed using an anti‐Iba‐1 antibody, was also unchanged by CKD‐504 in ADLP^APT^ mice (Figure S1k–m). However, we found that CKD‐504 did reduce tau pathology in ADLP^APT^ mice (Figure 2e‐j, Figure S2), a finding consistent with results obtained with AD patient‐iPSC‐derived brain organoids. CKD‐504 reduced the amount of total tau and phosphorylated tau (pT181, pS199, pS262, pT231, and pS396) in the sarkosyl‐soluble fraction at both doses (Figure 2e–g, Figure S2a–f). The amounts of total tau and phosphorylated tau (AT8, AT180, pS356, and pS262) in the sarkosyl‐insoluble fraction, which contains higher‐order filamentous aggregates of tau (Ren & Sahara, 2013), were also reduced by CKD‐504 (Figure 2h,i, Figure S2g–k). In addition, immunohistochemical analyses using Tau‐13, AT8, and AT180 antibodies consistently showed that CKD‐504 decreased the amounts of total and phosphorylated tau in ADLP^APT^ mice (Figure 2j, Figure S2l–q). Moreover, we confirmed that the decreased tau did not result from transcriptional regulation of tau (Figure S2r). These results demonstrate that a 1 mg/kg dose of CKD‐504 was sufficient to rescue tau pathologies, which might contribute to the rescue of memory impairment, but did not rescue Aβ pathology or neuroinflammation.

### 2.5 CKD‐504 rescues memory impairment and synaptic and tau pathologies in ADLP^APT^ mice in a therapeutic experimental paradigm

**Figure 3:**
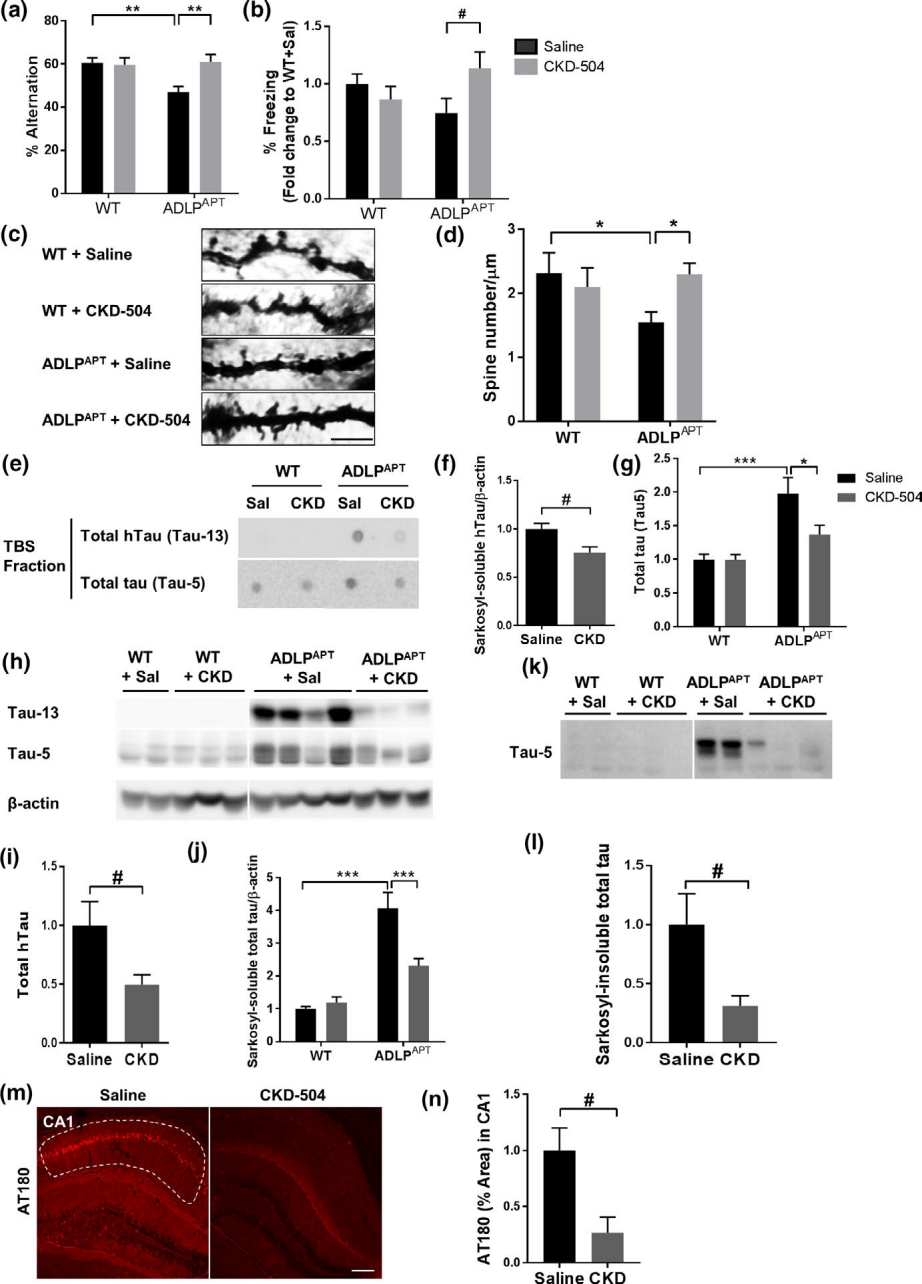
CKD‐504 recovered memory impairment and dendritic spine density, and reduced tau in therapeutic model of ADLP^APT^ mice. (a, b) Cognitive impairment was rescued by CKD‐504 injection for 2 months in 6.5‐month‐old ADLP^APT^ mice. Quantification of Y‐maze test (a) and CFC test (b) (*n* = 16–22). (c, d) CKD‐504 rescued dendritic spine density in the cortex. Representative images (c) and quantification (d) of cortical spines (*n* = 16–29 dendrites from 3 mice per group). (e‐g) Representative images (e) and quantification (f, g) of dot blot showing that CKD‐504 reduced total tau (*n* = 5–6). (h–l) Sarkosyl‐soluble and sarkosyl‐insoluble tau proteins were decreased by CKD‐504. Representative immunoblot images and quantification of tau from sarkosyl‐soluble fraction (h‐j) and sarkosyl‐insoluble fraction (k, l) of hippocampal lysates from ADLP^APT^ mice (*n* = 5). Representative images and quantifications of phosphorylated tau are in Figure S3. (m, n) AT180 immunoreactivity was reduced in the hippocampus. Representative images (m) and quantification (n) (*n* = 10, 5). Scale bar: 100 μm. Data are presented as means ± *SEM*. Two‐way ANOVA followed by Bonferroni post hoc test. *p < .05, **p < .01, ***p < .001. Student's *t* tests. *#p* < .05. CKD, CKD‐504; Sal, Saline

We investigated whether CKD‐504 exerts therapeutic efficacy after the appearance of memory impairment. Because ADLP^APT^ mice show cognitive impairment from 6‐month‐old (Kim et al., 2018), we employed a therapeutic paradigm with 6.5‐month‐old ADLP^APT^ mice. Those mice were injected with CKD‐504 for 2 months. In this experimental paradigm, CKD‐504 rescued memory impairment in ADLP^APT^ mice, as assessed by Y‐maze and contextual fear conditioning tests (Figure 3a,b). PSD95 level followed the same trends as memory recovery, showing tendency of restoration measured by Western blotting (Figure S3a,b). Dendritic spine density, measured by Golgi–Cox staining, was also significantly restored (Figure 3c,d). Although Aβ pathologies and neuroinflammation, analyzed using ELISA and immunohistochemistry, were not changed by CKD‐504, tau pathologies were reduced (Figure S3c–h, Figure 3e–n). Specifically, Tris‐buffered saline (TBS)‐fractionated tau, which includes both sarkosyl‐soluble and sarkosyl‐insoluble tau, was reduced by CKD‐504 (Figure 3e–g). CKD‐504 also reduced the amounts of total and phosphorylated tau in sarkosyl‐soluble and sarkosyl‐insoluble fractions (Figure 3h–l, Figure S3i–p). Moreover, the intensity of AT180 staining in immunohistochemical analyses was consistently decreased by CKD‐504 (Figure 3m,n). These data show that CKD‐504 rescues memory impairment, synaptic pathology, and tau pathologies, not only in the preventive model but also in the therapeutic model. Because CKD‐504 exerts therapeutic efficacy when administered at any stage of the disease, it might be a potential therapeutic drug for AD.

### 2.6 CKD‐504 potentiates proteasomal degradation of tau by changing their protein interactome to a chaperone network

**Figure 4:**
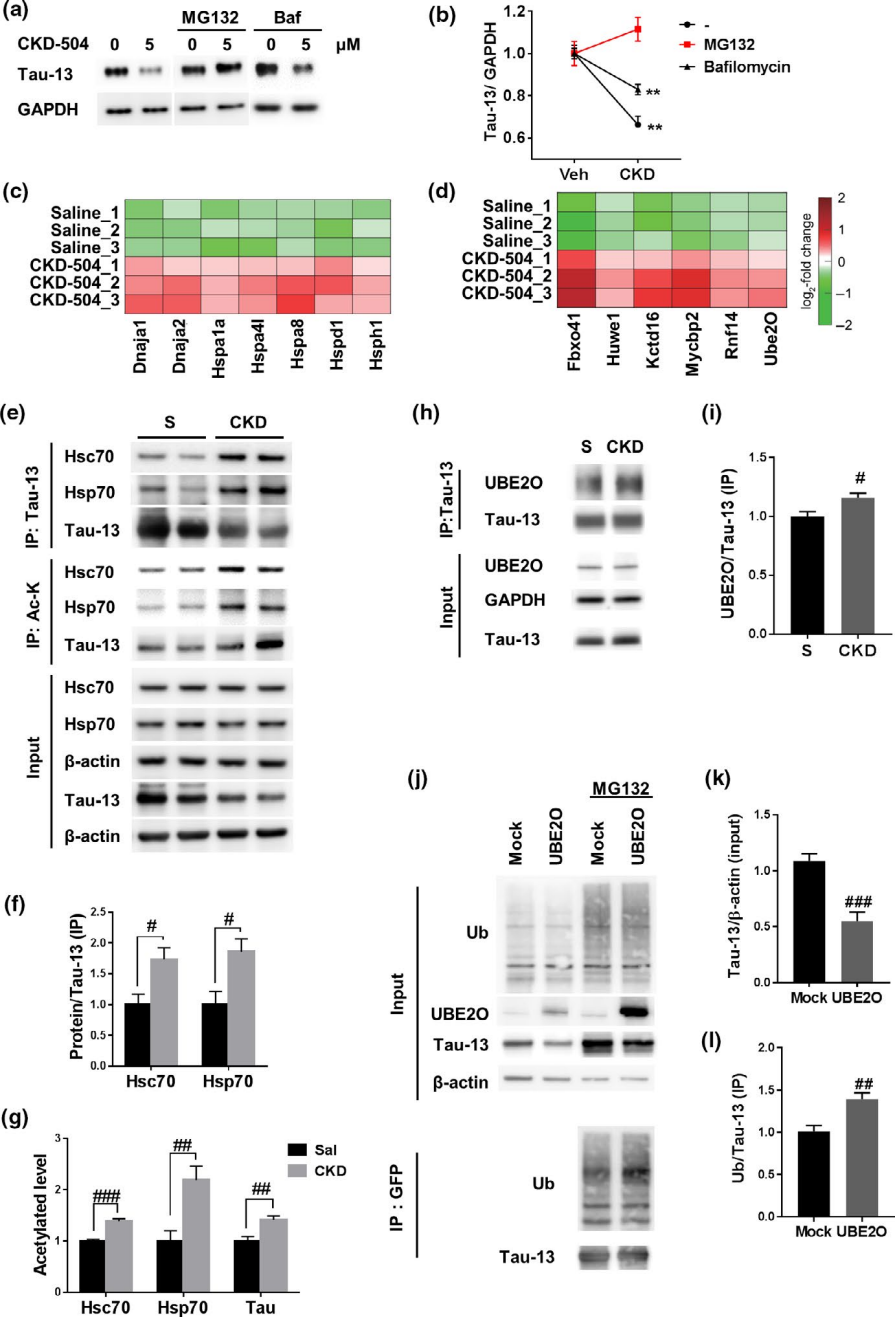
CKD‐504 potentiated proteasomal degradation of tau by changing tau protein interactome to chaperone network proteins such as heat shock proteins and E3 ligases. (a, b) Degradation of tau by CKD‐504 is mediated by proteasomal degradation in AD patient‐derived brain organoids. Brain organoids were treated with 20 μM of MG132 or 5 nM of bafilomycin for 4 hr. Representative images (a) and quantification (b) (*n* = 3). (c, d) The heat map represents log_2_ fold changes of intensities reflecting interaction strength, which were measured by mass spectrometric analysis in CKD‐504‐treated samples relative to saline controls. The color bar shows a gradient of log_2_ fold changes. (e–g) CKD‐504 increased acetylation and interactions between tau and chaperones in ADLP^APT^ mice. Human tau and acetylated proteins were immunoprecipitated by Tau‐13 antibodies and anti‐acetyl lysine antibody‐conjugated beads, respectively. Representative images (e) and quantification (f, g) (*n* = 3–5). (h, i) CKD‐504 increased the interaction between tau and UBE2O in ADLP^APT^ mice. Human tau was immunoprecipitated by Tau‐13 antibodies (*n* = 4). (j‐l) UBE2O ubiquitinated and reduced tau. Tau overexpressed HT22 cells were transfected with mock or UBE2O (MG132: 5 μM, 24 hr). Representative images (j) and quantification (k, l) (*n* = 5, independent experiments). Data are presented as means ± *SEM*. One‐way ANOVA followed by Bonferroni post hoc test. **p < .01. Student's *t* tests. *#p* < .05, *##p* < .01, *###p* < .001. Ac‐K, acetylated lysine; Baf, bafilomycin; CKD, CKD‐504; S, saline; Ub, ubiquitin

Based on our major finding that inhibition of HDAC6 by CKD‐504 reduced pathological tau, we next sought to investigate the underlying mechanisms. First, we examined whether HDAC6 regulated the turnover of tau in vitro. To measure the turnover rate of tau, we co‐transfected HT22 cells with human mutant tau (P301L) and HDAC6 plasmids and then treated these cells with cycloheximide (CHX), to inhibit de novo protein synthesis (Figure S4a,b). HT22 cells, transfected with human mutant tau (P301L) alone, were treated with CKD‐504 and CHX (Figure S4c,d). The tau degradation rate was decreased in cells overexpressing HDAC6 compared with mock‐transfected cells and was increased by CKD‐504 compared with vehicle. These results show that HDAC6 negatively regulates the degradation rate of tau. Next, because tau is degraded by both the UPS and ALS (Lee et al., 2013), we investigated which of these two systems is involved in the CKD‐504‐induced degradation of tau in AD brain organoids, P301L‐tau transfected HT22 cells, and ADLP^APT^ mice. We observed that CKD‐504 reduced tau in AD brain organoids treated with vehicle and lysosomal inhibitor, but failed to degrade tau in the presence of proteasomal inhibitor, MG132, showing effects of tau degradation with CKD‐504 are mediated by proteasomal degradation pathway (Figure 4a,b). To confirm this result in vitro, we used several different inhibitors in HT22 cells. Consistent with the results in human brain organoids, CKD‐504 accumulated tau in HT22 cells in the presence of MG132, but not in the presence of lysosome or autophagy inhibitors (Figure S4e,f). In this overexpression system of HT22 cells, because accelerated degradation rate by CKD‐504 might be insufficient for overcoming expression rate of overexpressed tau, CKD‐504 could not reduce tau level in the vehicle. Therefore, accumulated tau by CKD‐504 by MG132 indicates that CKD‐504 promotes proteasomal degradation of tau. Moreover, ubiquitinated tau was increased by CKD‐504 in the presence of the proteasome inhibitor in HT22 cells (Figure S4g). In the same vein, autophagy‐lysosomal flux was not rescued by CKD‐504 in HT22 cells and ADLP^APT^ mice, as evidenced by the absence of changes in ALS‐related proteins (Figure S5a–h). These data suggest that CKD‐504 potentiates proteasomal degradation of tau.

Ubiquitination and proteasomal degradation of proteins are regulated by a chaperone network that includes heat shock proteins and E3 ligases (Vilchez, Saez, & Dillin, 2014). Because CKD‐504 did not affect proteasome activity (Figure S5i), we hypothesized that CKD‐504 regulates interactions between tau and chaperones or co‐chaperones. To test this hypothesis, we performed a tau‐interactome analysis by mass spectrometry using saline‐ or CKD‐504‐injected ADLP^APT^ mouse brain samples co‐immunoprecipitated (co‐IPed) with Tau‐13 antibody. We identified 265 differentially interacting proteins in the CKD‐504 group compared with the saline group (*t* test FDR <0.05, absolute log_2_ fold change ≥0.58) (Tables S1 and S2). Several unexpected E3 ligases (Mycbp2, UBE2O, RNF14, Huwe1) which have not been reported to targeting tau were shown to exhibit increased interactions with tau following treatment with CKD‐504. An enrichment analysis of gene ontology biological processes (GOBPs) revealed that among the tau interactors that showed increased interactions in response to CKD‐504 included a significant number of microtubule‐associated proteins (Figure S6), supporting the validity of our proteomic analysis. This analysis further showed that heat shock proteins were significantly enriched among tau interactors that showed increased interactions in response to CKD‐504 (Figure 4c,d). These heat shock proteins included members of the Hsp40 family (Dnaja1, Dnaja2), Hsp70 family (Hspa1a/Hsp70, Hspa4l, Hspa8/Hsc70), Hsp60 family (Hspd1), and Hsp110 family (Hsph1). These data indicate that CKD‐504 increases interactions between tau and chaperone network proteins, an effect that could be responsible for the observed proteasomal degradation of tau.

**Figure 5:**
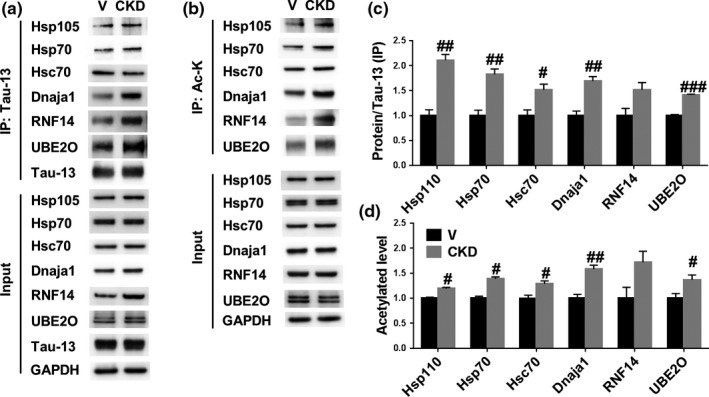
CKD‐504 increased interaction and acetylation of chaperone network proteins in AD patient‐iPSC‐derived brain organoids. (a‐d) CKD‐504 increased acetylation and interactions between tau and chaperone network proteins in AD brain organoids. Human tau and acetylated proteins were immunoprecipitated by Tau‐13 and anti‐acetyl lysine antibody‐conjugated beads. Representative images (a, b) and quantification (c, d). *n* means independent number of organoids. Data are presented as means ± *SEM*. Student's *t* tests. *#p* < .05, *##p* < .01, *###p* < .001. Ac‐K, acetylated lysine; CKD, CKD‐504; Veh, vehicle

Next, we validated the differentially increased interactions between tau and chaperones and co‐chaperones induced by CKD‐504. It has been reported that Hsc70, Hsp70, and E3 ligases interact with tau and promote proteasomal degradation of tau (Shimura, Schwartz, Gygi, & Kosik, 2004; Wang, Tan, Lu, Yu, & Tan, 2014). Thus, to determine whether CKD‐504 alters interactions among tau, Hsc70, and Hsp70, we immunoprecipitated brain lysates with a Tau‐13 antibody and probed immunoprecipitates with antibodies against Hsc70 and Hsp70. As expected, interactions among Hsc70, Hsp70, and tau were increased in ADLP^APT^ mice injected with CKD‐504 compared with saline‐injected mice (Figure 4e–g). Moreover, interaction with tau and UBE2O, an E3 ligase newly identified targeting tau in this study, was increased in ADLP^APT^ mice injected with CKD‐504 compared with saline‐injected mice (Figure 4h,i). Therefore, these data demonstrate that CKD‐504 regulates interactions between tau and chaperone network proteins. We further determined the activity of UBE2O as an E3 ligase of tau by overexpressing UBE2O in HT22 cells. UBE2O overexpressed HT22 cells showed a decreased level of tau and increased ubiquitination of tau (Figure 4j–l). From these results, UBE2O might be a novel E3 ligase targeting tau. Because HDAC6 regulates acetylation of proteins, we considered the possibility that CKD‐504 increased acetylation of tau and tau interactors. To detect acetylation levels of these proteins, we immunoprecipitated lysates with anti‐acetyl lysine affinity beads and then probed beads with the indicated antibodies. CKD‐504 increased acetylation of Hsc70, Hsp70, and tau in ADLP^APT^ mice (Figure 4e–g). Consistent with in vivo data, interactions among tau and chaperones (Hsp105, Hsp70, Hsc70, Dnaja1), E3 ligases (RNF14, UBE2O), and also acetylation of chaperones and E3 ligases were increased by CKD‐504 in brain organoids (Figure 5). These results suggest that CKD‐504 increases interactions among chaperone network proteins and tau by increasing acetylation of tau and several chaperone network proteins, which leads to proteasomal degradation of tau (Figures 2, 3, and 4a). Thus, our findings demonstrate that CKD‐504 facilitates proteasomal degradation of tau by regulating the acetylation level of tau and chaperone network proteins.

### 2.7 Acetylation at K274, K290, K321, and K353 of tau regulates tau turnover rate

**Figure 6:**
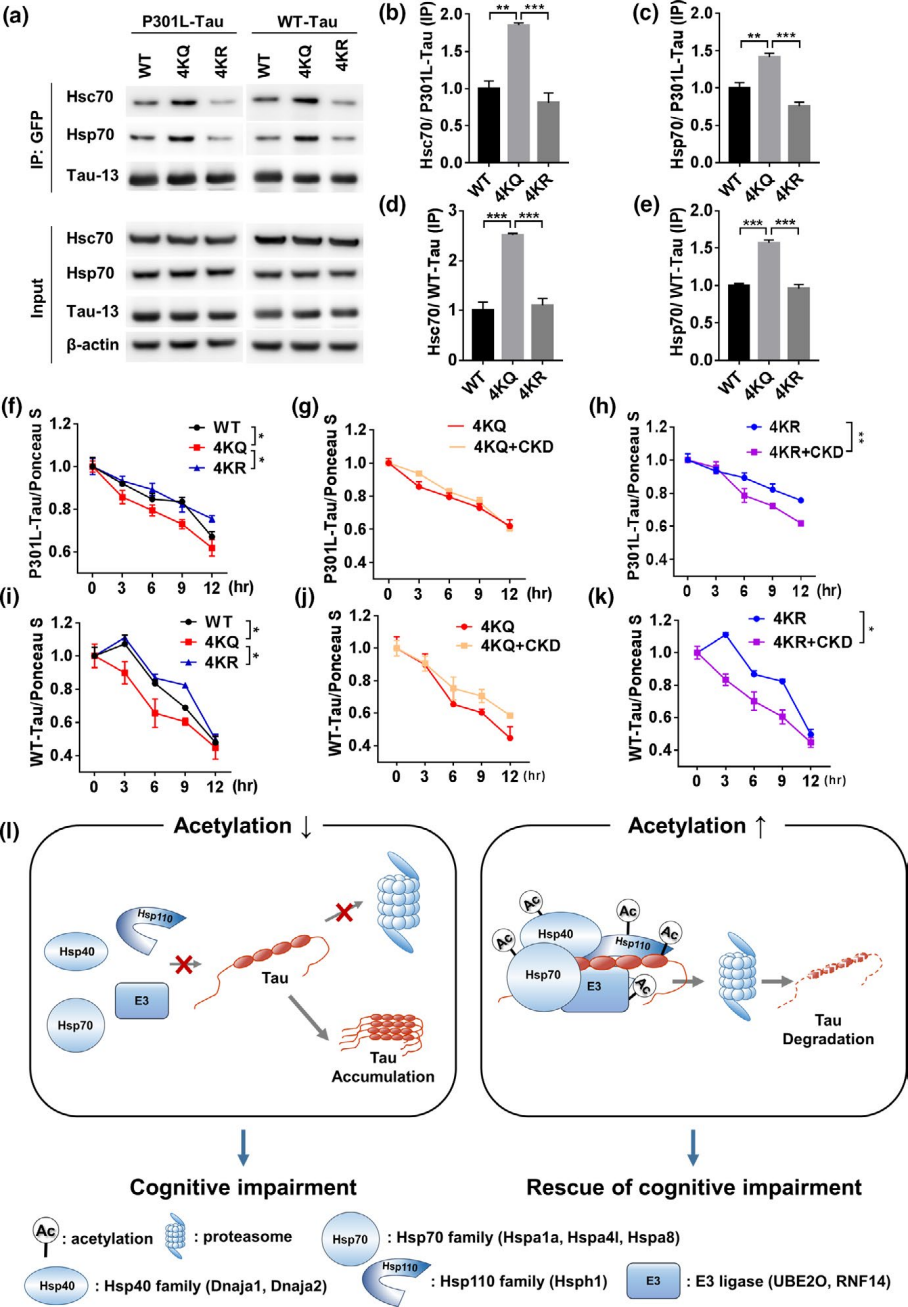
Acetylation on K274, K290, K321, and K353 of tau regulates interaction with chaperones and degradation rate of tau. (a–k) Acetyl‐mimic and acetyl‐silencing mutations on K274, K290, K321, and K353 of P301L and WT tau regulated interactions with tau and chaperones in HT22 cells. Representative immunoblot images (a) and quantification of mutations in P301L tau (b, c) and in WT tau (d, e) (*n* = 3, independent experiments). Data are presented as means ± *SEM*. One‐way ANOVA followed by Bonferroni post hoc test. *p < .05, **p < .01, ***p < .001. (f–k) Quantitative graphs of altered degradation rate of tau by acetyl‐mimic and acetyl‐silencing mutations in tau and CKD‐504. Representative immunoblot images in Figure S7h. Data are presented as means ± *SEM*. Analysis of covariance (ANCOVA). *p < .05, **p < .01. (l) Schematic diagram of the mechanism of tau degradation by CKD‐504. In AD, as overall level of acetylation is down‐regulated, interactions of tau with chaperones and E3 ligases may be insufficient to degrade pathologic tau. Consequently, tau is hardly degraded by UPS and becomes aggregated. CKD‐504, an HDAC6 inhibitor, increases acetylation levels of tau and several chaperone network proteins and E3 ligases and increases interactions with tau, resulting in promoting proteasomal degradation of tau. Finally, reduced tau by CKD‐504 leads to rescue of cognitive impairment in AD model mice

As previously reported, CKD‐504 regulates tau acetylation by inhibiting HDAC6 (Carlomagno et al., 2017; Cook et al., 2014; Ding et al., 2008). However, the acetylation sites that contribute to proteasomal degradation of tau were unclear. It has been reported that tau acetylation at K274, K290, K321, and K353—the acetylation sites regulated by HDAC6—decreases heparin‐induced aggregation of tau (Carlomagno et al., 2017). In addition, ^275^VQII^278^ and ^306^VQIV^309^ motifs in the microtubule‐binding domain of tau are known to mediate tau association with Hsc70 and Hsp70 (Sarkar, Kuret, & Lee, 2008). K274, one of the four aforementioned acetylation sites, is located immediately before the ^275^VQII^278^ motif. Therefore, we hypothesized that acetylation of tau at K274, K290, K321, and K353 regulates interactions among tau and chaperone network proteins and degradation of tau. To test this hypothesis, we constructed acetyl‐mimic or acetyl‐silencing mutants by substituting two lysine sites (K274, K321), which are the most effective anti‐aggregation sites, and all four lysine sites (K274, K290, K321, and K353) with glutamine (2KQ, 4KQ) or arginine (2KR, 4KR), respectively, in both normal tau indicated by WT tau and pathological tau such as P301L‐tau. Subsequently, we tested the interactions among WT tau or mutant tau and the representative chaperone network proteins in HT22 cells. Interaction between Hsp70 and tau was increased in 2KQ mutants, but not in 2KR mutants (Figure S7a–c). However, turnover rates of tau were not altered in both 2KQ and 2KR mutants (Figure S7d–g). As 2KQ mutants showed increased interaction with Hsp70 but did not change the turnover rate of tau, we subsequently evaluated effects of 4KQ and 4KR mutants, which are additionally modulated acetylation sites. 4KQ mutants showed increased interactions with Hsc70 and Hsp70, whereas interactions of 4KR mutants with those chaperones were unaltered compared with WT, which is similar to 2KQ/2KR mutants (Figure 6a–e). However, turnover rates of 4KQ mutants in HT22 cells were increased compared with WT and 4KR mutant (Figure 6f,j, Figure S7h). These results suggest that all four acetylation sites that we have tested are important for promoting degradation of tau.

To determine whether acetylation on K274, K290, K321, and K353 of tau is responsible for regulating tau degradation by CKD‐504, we observed the effect of CKD‐504 on 4KQ or 4KR mutants (Figure 6g,h,j,k, Figure S7a). CKD‐504 did not accelerate the degradation rate of tau in the 4KQ mutant, which means that acetylation on the four residues of tau is responsible for regulating tau degradation. However, CKD‐504 increased the degradation rate of tau in 4KR mutant. It might be because CKD‐504 increases acetylation of chaperones, E3 ligases, and other HDAC6‐regulated sites on tau. These results suggest that acetylation on the four lysine residues of tau is sufficient for promoting tau degradation.

Collectively, our findings demonstrate that acetylation of tau on K274, K290, K321, and K353 sufficiently regulates the turnover rate of tau by recruiting Hsc70 and Hsp70 to tau.

## 3 DISCUSSION

In the present study, we demonstrated the therapeutic efficacy of CKD‐504, a novel and selective HDAC6 inhibitor that shows high penetration in the brain. CKD‐504 rescued memory impairment and synaptic pathology in ADLP^APT^ mice, which exhibit both Aβ and tau pathologies similar to patients with AD, primarily by reducing pathological tau. In addition, CKD‐504 reduced pathological tau in AD patient‐iPSC‐derived brain organoids, suggesting that CKD‐504 might efficiently regulate tau in the human brain. CKD‐504 also regulated acetylation of tau and the chaperone proteins such as Hsc70 and Hsp70, enhancing the interactions among these proteins as well as the recruitment of the novel tau E3 ligases (UBE2O and RNF14), followed by ubiquitination and proteasomal degradation of tau (Figure 6l). Moreover, we revealed that acetylation on K274, K290, K321, and K353 of tau is involved in proteasomal degradation of tau by CKD‐504. Taken together, our findings elucidate the mechanism by which the HDAC6 inhibitor, CKD‐504, causes tau degradation, thereby establishing its therapeutic potential.

A recent study reported that another HDAC6 inhibitor, MPT0G211, increased acetylation of Hsp90 and ubiquitination of phosphorylated tau (Fan, Huang, Liou, & Yang, 2018). However, the precise mechanism of tau ubiquitination was unclear. In the current study, we demonstrated that CKD‐504 recruited the E3 ligases to tau by increasing acetylation of tau and several chaperone network proteins such as Hsp105, Hsc70, Hsp70, Dnaja1, RNF14, and UBE2O, thereby enhancing complex formation. In addition, we identified UBE2O and RNF14 as novel E3 ligases of tau and showed that CKD‐504 increased the interaction of these proteins with tau in mouse brain and patient‐derived human brain organoids. The specific role of each of these proteins will be examined in future studies. However, based on previous reports about Hsp40 and Hsp110 family (details in extended discussions), CKD‐504 may decrease tau aggregates by inhibiting hyperphosphorylation and aggregation or by promoting disaggregation; it may also promote tau clearance by regulating the aforementioned chaperones and co‐chaperones. Therefore, we demonstrated for the first time that an HDAC6 inhibitor could orchestrate a chaperone network rather than merely a single component.

We also determined that acetylation of tau on K274, K290, K321, and K353 plays an important role in the formation of complexes with chaperones and degradation of tau. Because CKD‐504 has no additive effects on the degradation of tau in 4KQ mutant, the acetylation on the four lysine residues of tau is sufficient for promoting the degradation of tau by CKD‐504. In addition, the results of 2KQ mutants, in which the interaction between Hsp70 and tau was increased, whereas turnover rates of tau were not altered, also show the four acetylation sites are important for promoting tau degradation. Since 4KQ mutants regulate both interaction between chaperones and tau and turnover rates of tau, the results of double mutants suggest that the role of acetylation on K274 and K321 of tau is only regulating the interaction with chaperones such as Hsp70. It seems that other acetylation sites—K290 and K353—might be responsible for an additional mechanism for tau degradation such as binding with E3 ligases. Indeed, ^275^VQII^278^ and ^306^VQIV^309^ motifs of tau are known to mediate tau association with Hsc70 and Hsp70 (Sarkar et al., 2008), which implies that K274 and K321 are responsible for interacting with Hsp70. In addition, UBE2O, one of the E3 ligases that is validated in this study, was known to bind with VLI patch (Mashtalir et al., 2014). K290 and K353 are located within the similar sequence with VLI patch motif on microtubule‐binding regions (244–367 a.a.) of tau. Therefore, acetylation on K290 and K353 of tau might be critical for binding E3 ligases. Collectively, all four sites that we have tested are important acetylation sites for tau interaction with chaperones and E3 ligases, which promotes the degradation of tau.

CKD‐504 rescued memory impairment in ADLP^APT^ mice in both preventive and therapeutic experimental paradigms, in which mice were injected with CKD‐504 before or after memory impairment appeared, respectively. This indicates that CKD‐504 not only protects against the development of cognitive dysfunction, but it also rescues impaired cognitive function in ADLP^APT^ mice. Given that many AD clinical trials, including those involving AD dementia patients, have failed and recent AD clinical trials tend to recruit patients with preclinical and prodromal AD (Anderson et al., 2017; Cummings, Lee, Mortsdorf, Ritter, & Zhong, 2017), the ability of CKD‐504 to rescue memory impairment even in a therapeutic experimental paradigm suggests that this agent has strong potential for treating AD. In addition, other therapeutic effects of CKD‐504 and a prolonged effect after washout of CKD‐504 support the strong therapeutic potential of CKD‐504 (Figure S8, extended discussion). Higher‐order aggregates of tau are known to be degraded by the ALS, whereas buffer‐soluble tau is cleared by the UPS (Lee et al., 2013). Higher‐order aggregates of tau, which are present in a sarkosyl‐insoluble fraction (Ren & Sahara, 2013) from ADLP^APT^ mice, were reduced by CKD‐504, despite the fact that CKD‐504 does not affect the ALS. It has been reported that increased acetylation of tau at HDAC6‐regulating sites, such as KXGS motifs and K274, inhibits aggregation of tau (Carlomagno et al., 2017). Therefore, a reduction in sarkosyl‐insoluble tau by CKD‐504 might have resulted from blocking tau aggregation by HDAC6 inhibition, followed by proteasomal degradation of soluble aggregates of tau. The HDAC6‐regulating sites, K274 and K280, are well‐known pathological acetylation sites of tau (Carlomagno et al., 2017; Irwin et al., 2012; Tracy et al., 2016). However, the apparent net effect of CKD‐504 on degrading tau may indicate that a number of acetylation sites are regulated by HDAC6 function together. Additionally, K174 and K281, two other reported pathological acetylation sites of tau, are not regulated by HDAC6 (Carlomagno et al., 2017; Min et al., 2015; Sohn et al., 2016).

In summary, we demonstrated that acetylation regulated by CKD‐504, a highly selective HDAC6 inhibitor with efficient BBB penetration, potentiated proteasomal degradation of tau by orchestrating a tau‐regulated chaperone network. Specifically, CKD‐504 increased acetylation of several chaperones and E3 ligases as well as tau itself, resulting in recruitment of the novel tau targeting E3 ligases such as UBE2O and RNF14 to tau, which lead to the rescue of synaptic pathology and memory impairment in ADLP^APT^ mice. These results allowed us to elucidate the precise mechanism of tau degradation by HDAC6 inhibition, in particular showing that acetylation of tau at K274, K290, K321, and K353 plays an important role in tau degradation. In addition, CKD‐504 showed therapeutic effects in both preventive and therapeutic experimental paradigms. Taken together, our results suggest that HDAC6 inhibitor could be a potent therapeutic drug candidate in AD that acts by modulating tau as well as a tau‐degrading chaperone network.

## 4 EXPERIMENTAL PROCEDURES

### 4.1 Animals and intraperitoneal injections

5XFAD mice (Tg6799; Jackson Laboratory, Stock#006554) and JNPL3 mice (TauP301L‐JNPL3; Taconic, Stock#2508 homozygote) are crossed to produce ADLP^APT^ mice. As background of JNPL3 mice is differ from 5XFAD mice, JNPL3 mice were backcrossed with B6SJL (C57BL/6 X SJL) mice before crossing with 5xFAD. Female WT and ADLP^APT^ mice were used for brain tissue analysis after CKD‐504 (Chong Kun Dang, South Korea) injection. We designed two models to determine the efficacy of CKD‐504 before or after memory impairment which appeared around 6‐month‐old ADLP^APT^ mice (Kim et al., 2018). For the preventive model, CKD‐504 (1 or 2.5 mg/kg) or saline were administered intraperitoneally to 4.5‐month‐old mice, twice a day, for four months. For the therapeutic model, 2.5 mg/kg of CKD‐504 or saline was administered intraperitoneally to 6.5‐month‐old mice, twice a day, for two months. Animals were treated and maintained in accordance with the Animal Care and Use Guidelines of Seoul National University, Seoul, Korea.

### 4.2 Behavioral tests

For Y‐maze test, a mouse was introduced to the middle of maze and allowed to freely explore the maze for 8 min. Spontaneous alternation was measured to analyze spatial memory function. To calculate percentage of spontaneous alternation, the number of sequential entries to different Y‐maze arms was divided by total arm entries. For contextual fear conditioning test (CFC), on day 1, a mouse was placed for a total of 5 min in an isolation cubicle with an electrifiable grid floor (Coulbourn, USA), with 0.55 mA foot shocks for 2 s each at 180 and 240 s. On day 2, all of the experiments were performed in the same conditions as day 1, without a foot shock. A mouse was allowed to freely explore the same chamber for 3 min, and the freezing behavior was analyzed by Freezeframe software (Coulbourn, USA).

### 4.3 Generation of human brain cortical organoids from hiPSCs

AD patient‐derived hiPSCs (participant no.: CW50039) and healthy control‐derived hiPSCs (participant no.: cw50071) were purchased from Coriell Institute for Medical Research (Camden, USA). Brain cortical organoids were generated using previously reported methods. Briefly, to form embryoid bodies (EBs), suspended iPSC colonies were incubated for 48 hr and the aggregated EBs were moved to Corning low attachment plates. After five days, the floating spheroids were transferred into neuronal medium. FGF2 and EGF were replaced with 100 μg/ml of BDNF and 100 μg/ml of NT3 on the twentieth day to promote neural differentiation, and the brain cortical organoids were then used for the experiments.

For details of methods, see Supporting Information.

## CONFLICT OF INTEREST

The authors declare no conflict of interests.

## AUTHOR CONTRIBUTIONS

HC, JY, and HJK designed the research, performed animal experiments such as injection, behavior tests, immunohistochemistry, tau fractionation, and Western blotting, and drafted the manuscript. HC and HJK also performed immunoprecipitation, CHX assay, proteasome activity assay, cloning, and brain organoid experiments. JY also carried out Golgi–Cox staining. WL, SC, JK, and HC contributed to animal experiments. SC and DH performed mass spectrometric analysis and bioinformatical analysis. J‐CP, DL, and S‐HH cultured brain cortical organoids. HS and CKL synthesized CKD‐504. JHJ and YJL performed HDAC enzymatic assays. KL and SK performed pharmacokinetics studies. CKL, YJL, SK, HS, YIC and KHR supervised developing process of CKD‐504 and served intellectual contribution. HC, JK, JK, and HC performed mitochondrial axonal transport analysis, and DCFDA and Fluo‐4 assays. IM‐J supervised the research, brought intellectual feedback, participated in interpretation of data, and drafted manuscript. All authors have read and approved the final manuscript.

## Data Availability

The data that support the findings of this study are available from the corresponding author upon reasonable request.
