# Supplementary figures and images for: Acetylation changes tau interactome to degrade tau in Alzheimer’s disease animal and organoid models
Source: Aging Cell. 2019 Nov 25;19(1):e13081. doi: 10.1111/acel.13081 (PMC6974726; doi:10.1111/acel.13081)

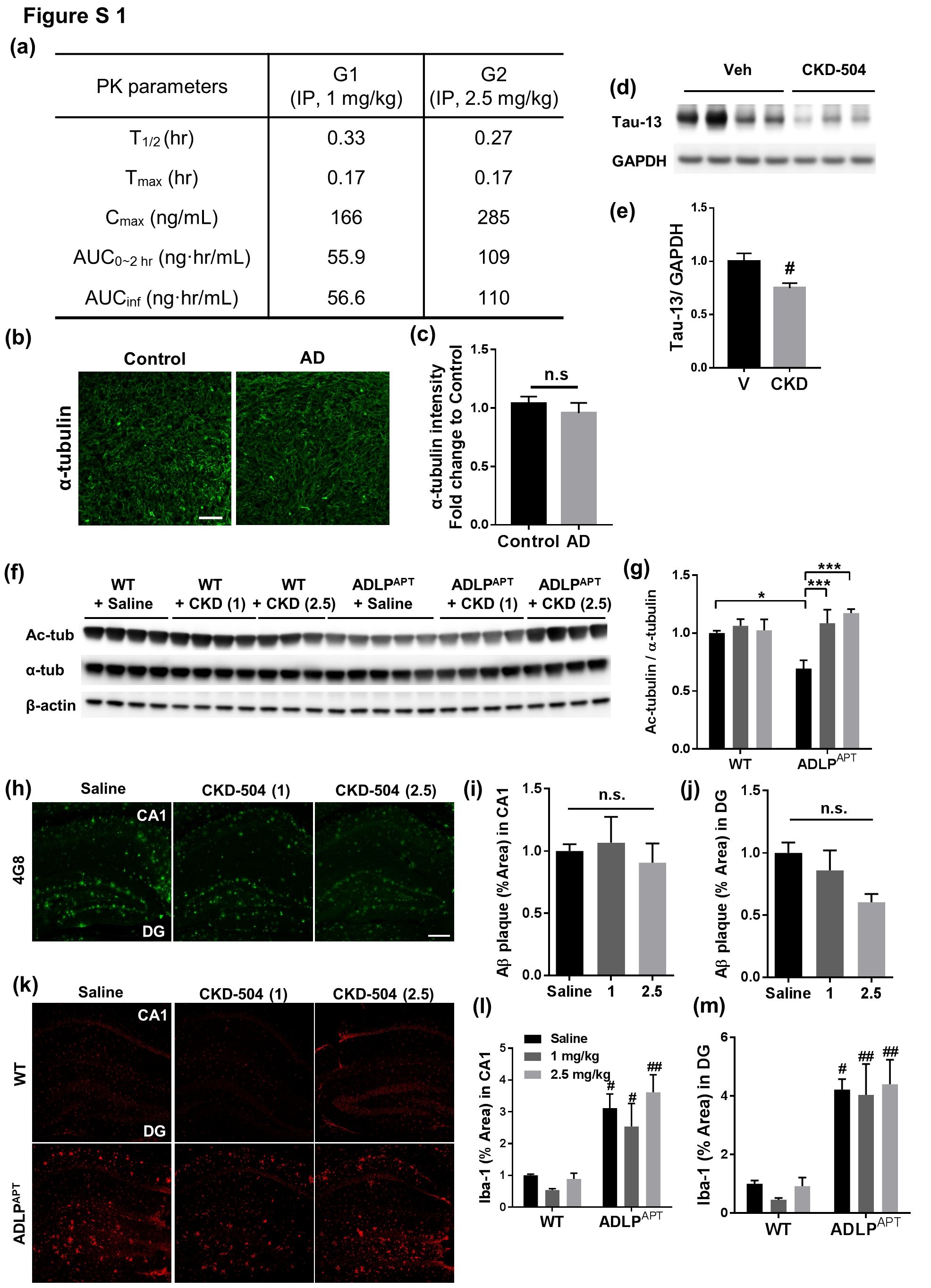

Supplement: Supplementary file 1 [file ACEL-19-e13081-s001.TIF]

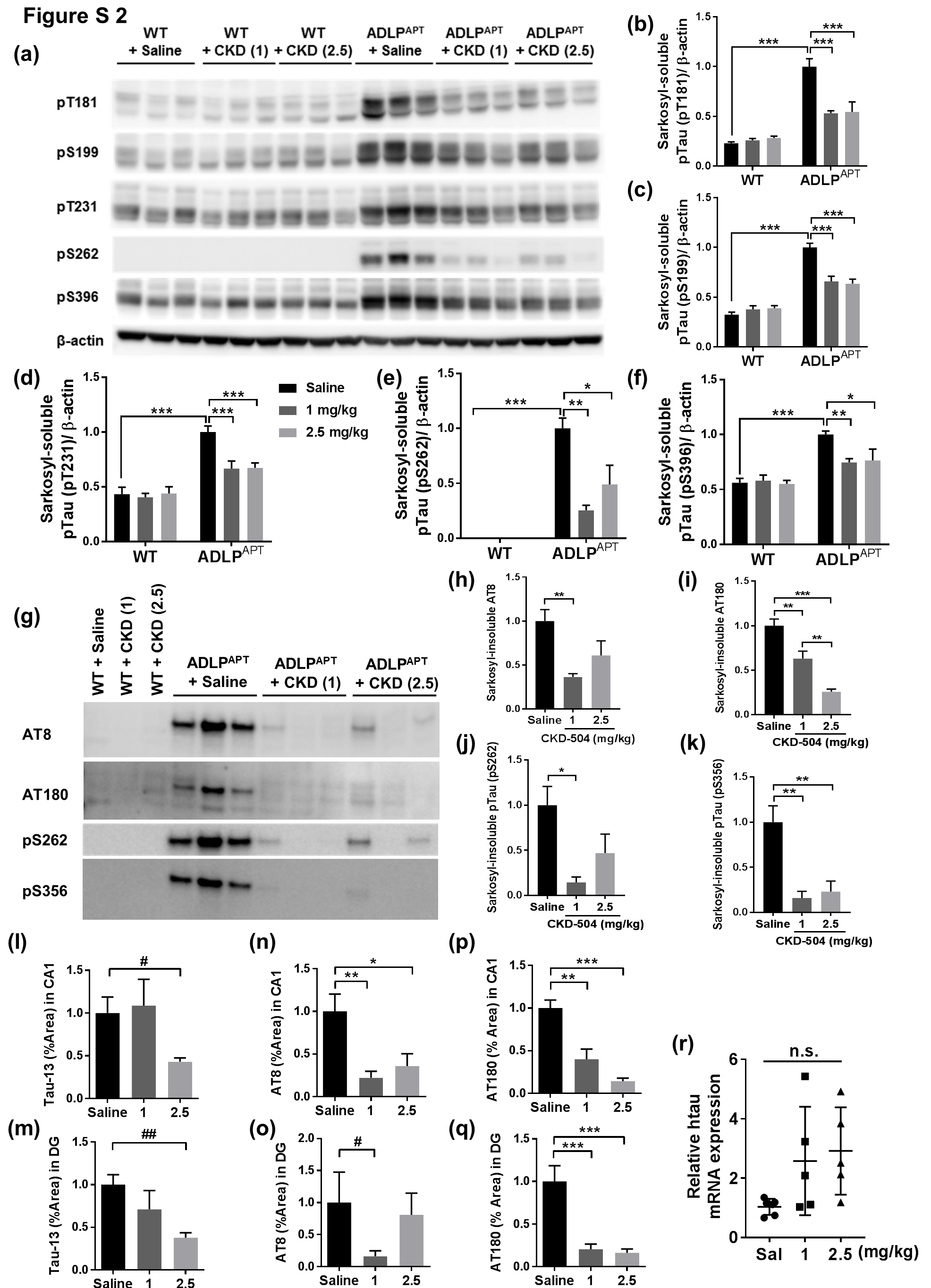

Supplement: Supplementary file 2 [file ACEL-19-e13081-s002.TIF]

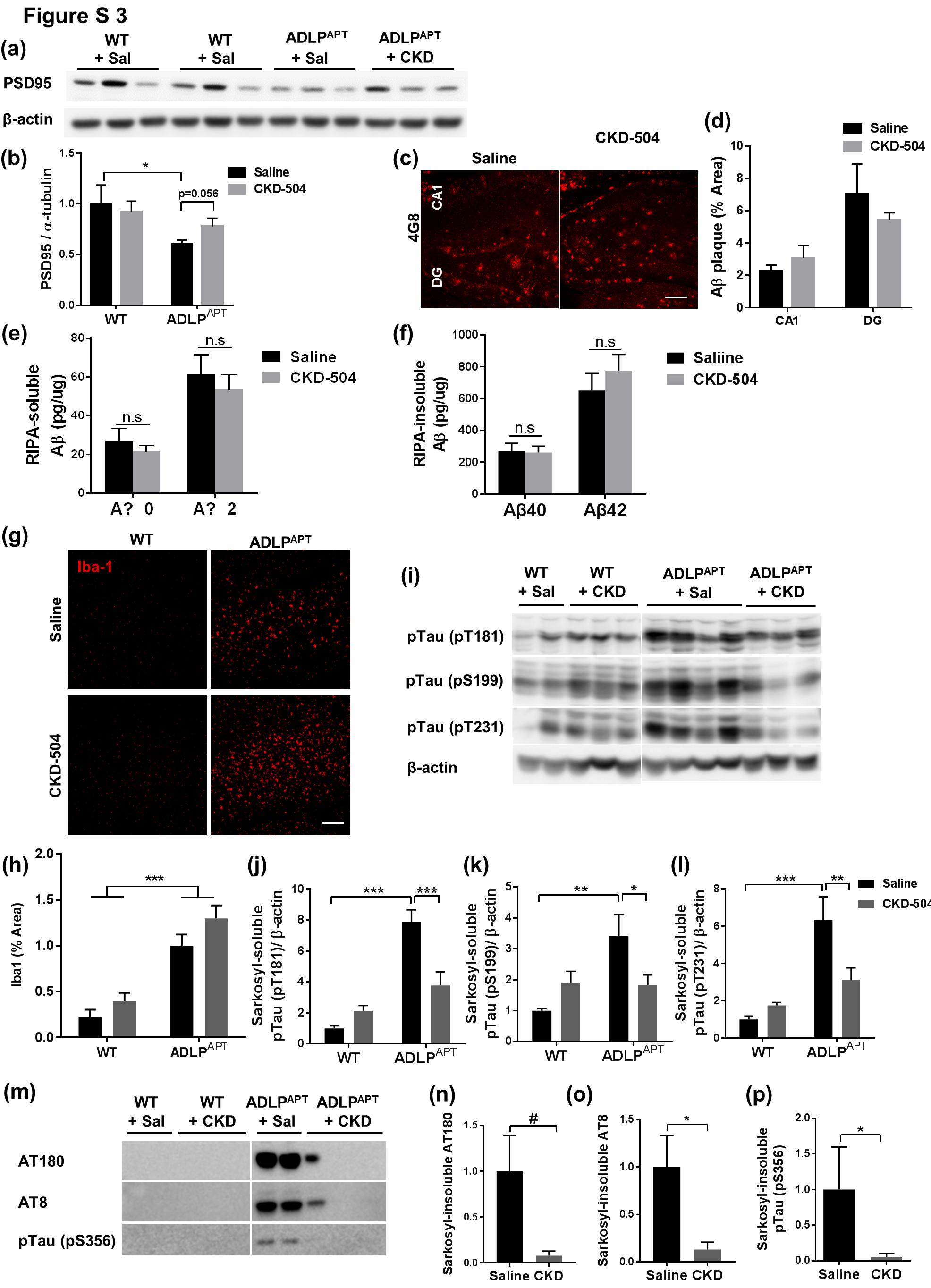

Supplement: Supplementary file 3 [file ACEL-19-e13081-s003.tif]

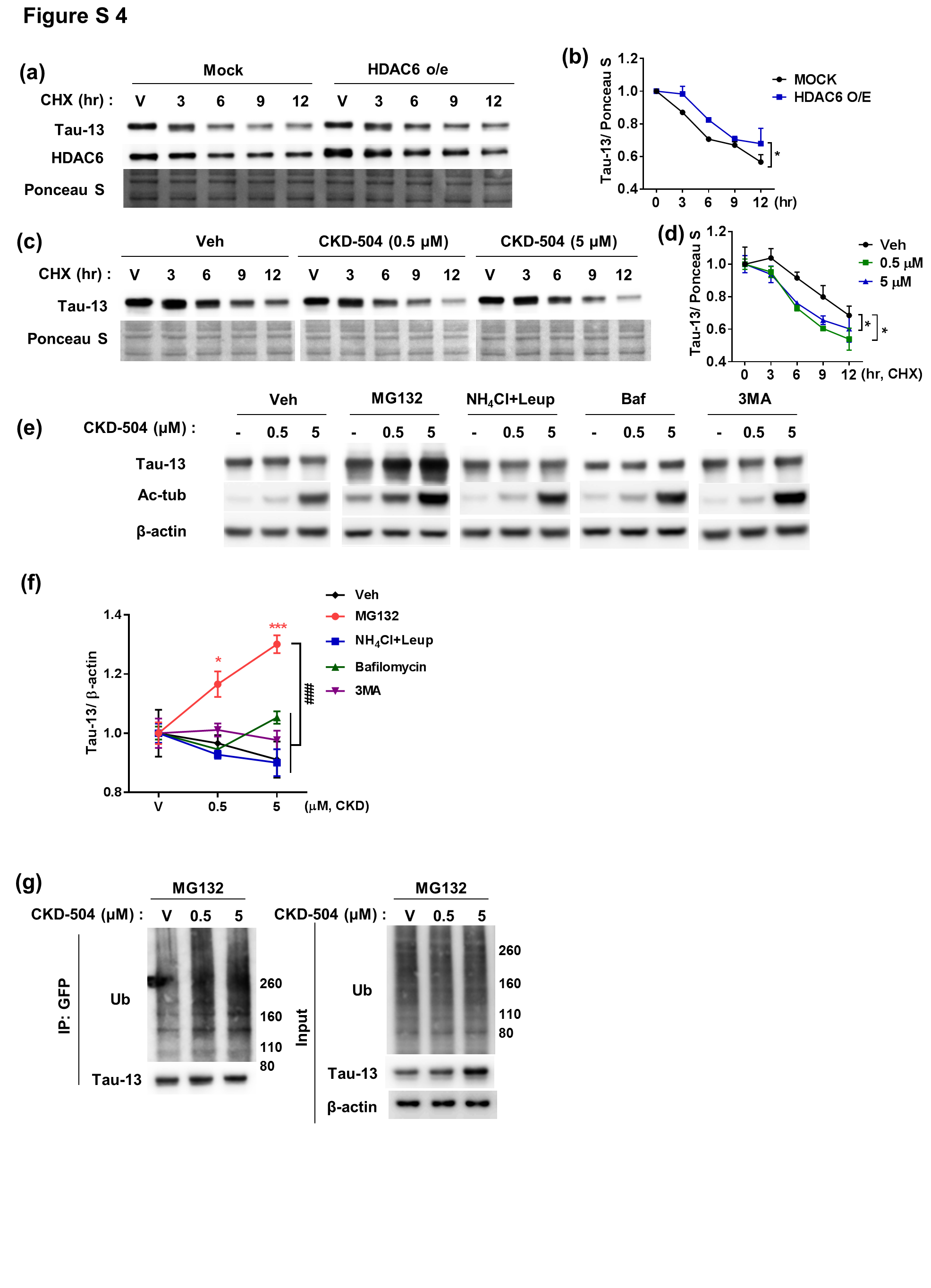

Supplement: Supplementary file 4 [file ACEL-19-e13081-s004.TIF]

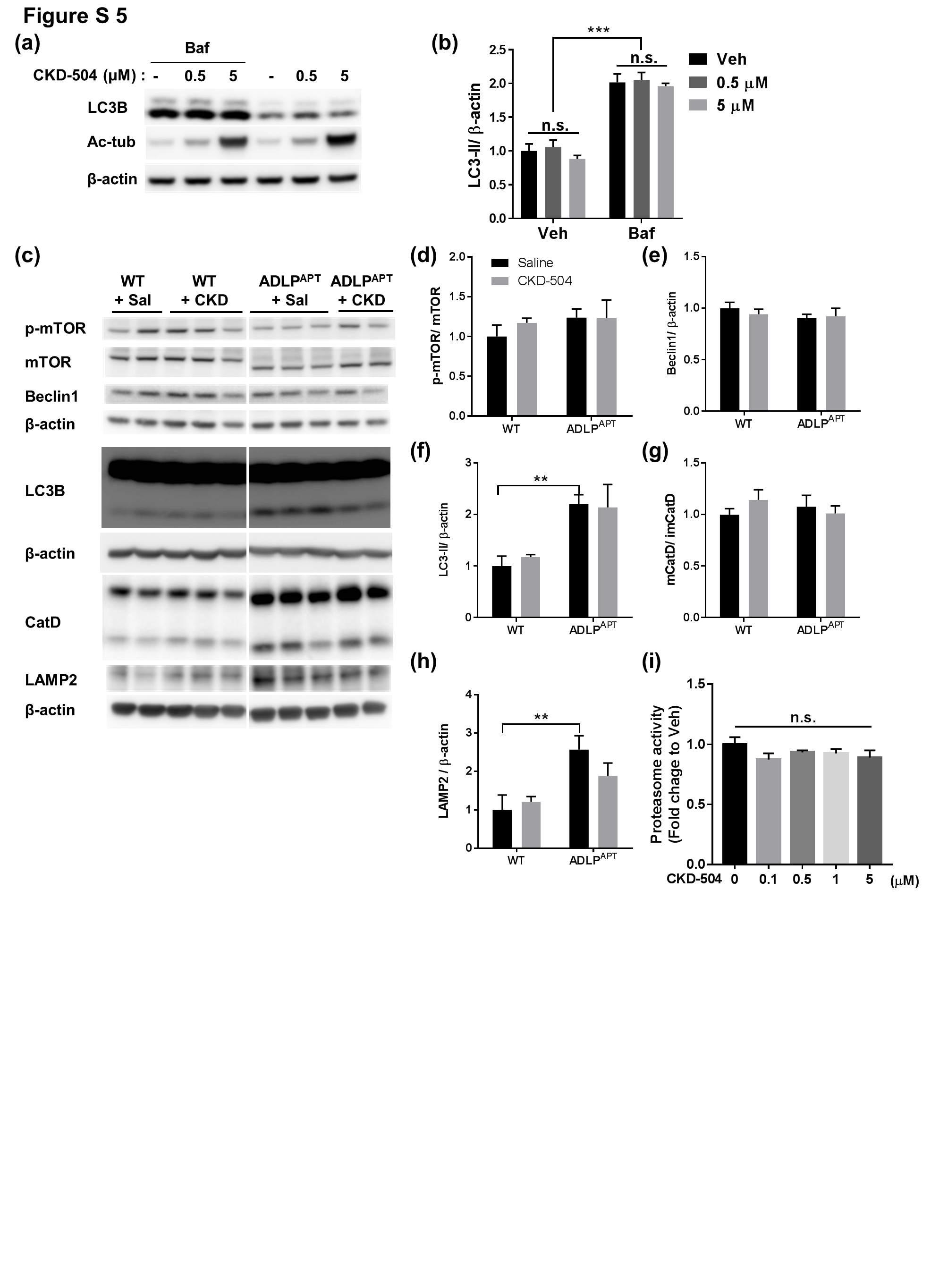

Supplement: Supplementary file 5 [file ACEL-19-e13081-s005.TIF]

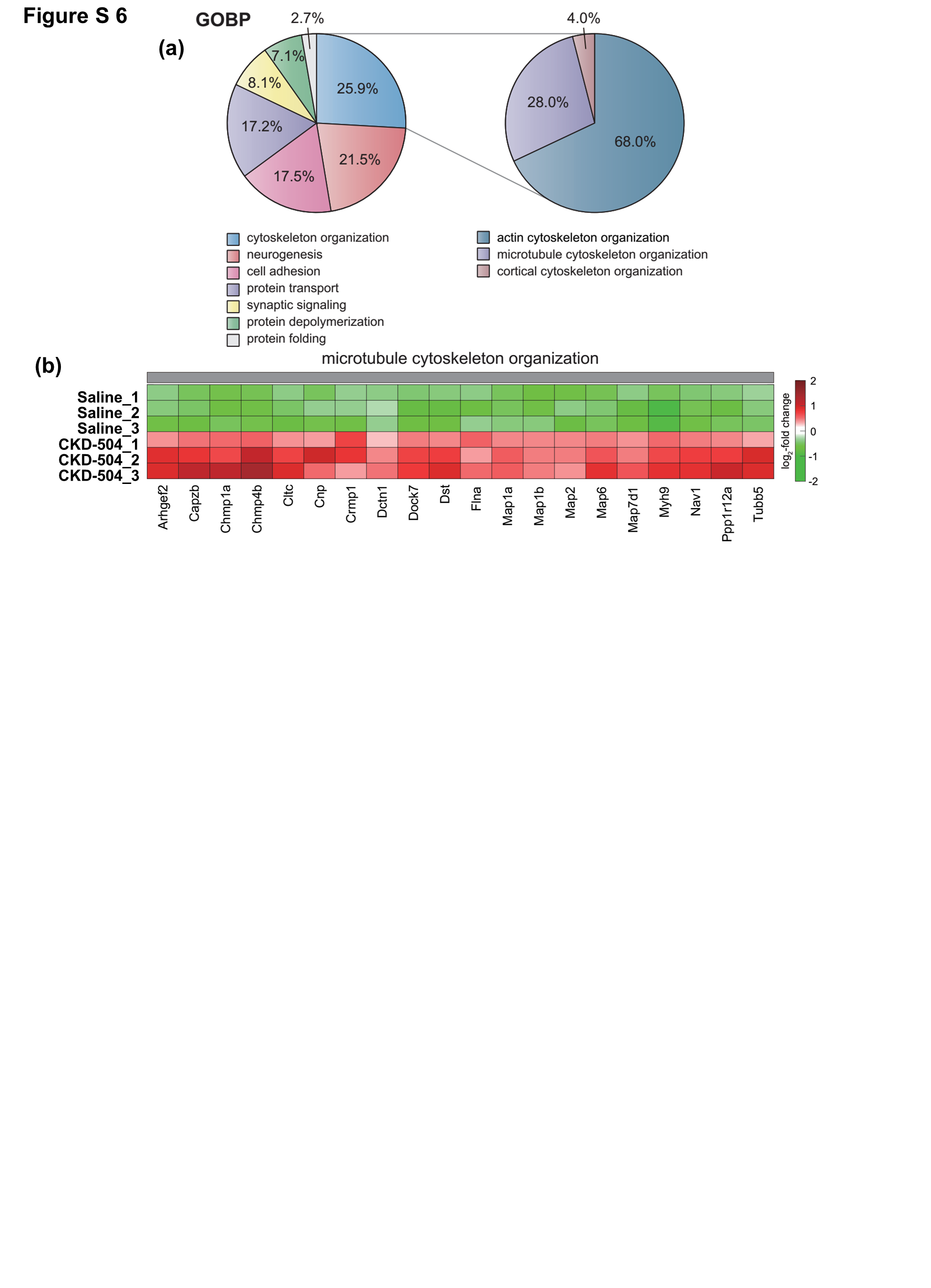

Supplement: Supplementary file 6 [file ACEL-19-e13081-s006.TIF]

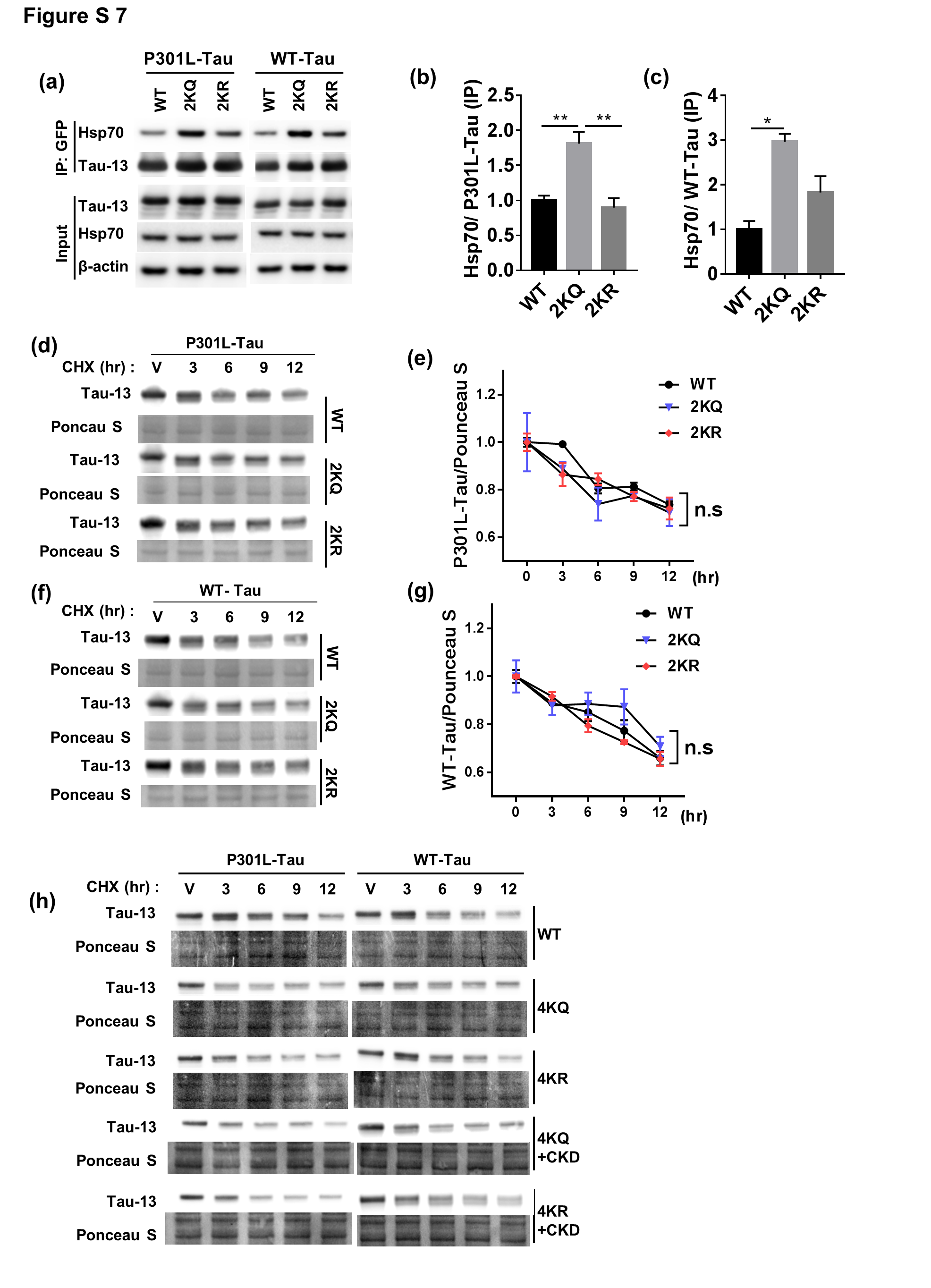

Supplement: Supplementary file 7 [file ACEL-19-e13081-s007.TIF]

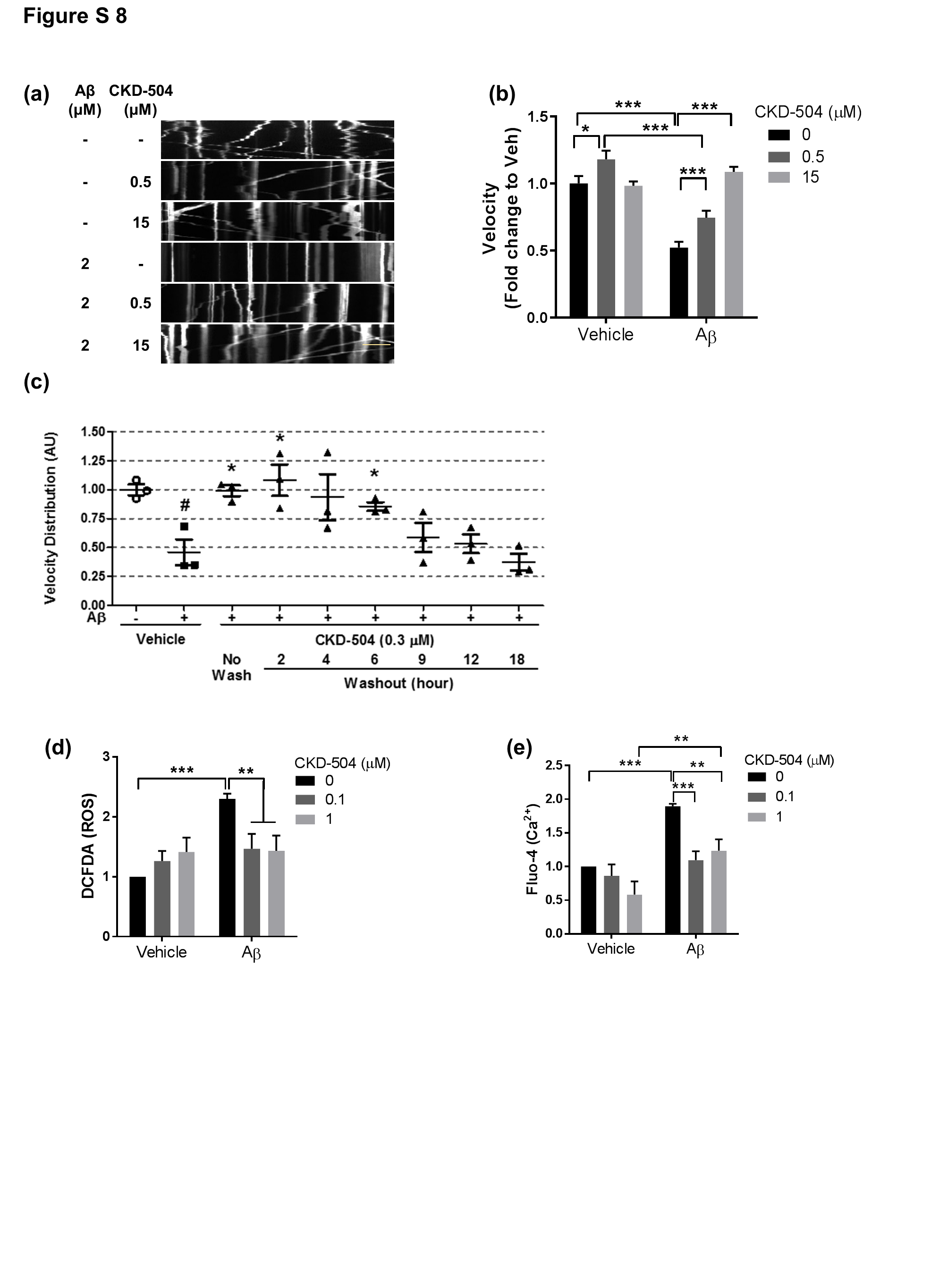

Supplement: Supplementary file 8 [file ACEL-19-e13081-s008.TIF]
