# Supplementary material for: Acetylation changes tau interactome to degrade tau in Alzheimer’s disease animal and organoid models
Source: Aging Cell. 2019 Nov 25;19(1):e13081. doi: 10.1111/acel.13081 (PMC6974726; doi:10.1111/acel.13081)
Supplement: Supplementary file 10 [file ACEL-19-e13081-s010.docx]

**SUPPORTING INFORMATION**

**Acetylation changes tau interactome to degrade tau in Alzheimer’s disease animal and organoid models**

**Authors:** Heesun Choi^1,#^, Haeng Jun Kim^1,#^, Jinhee Yang^1,5,#^, Sehyun Chae^2,3^, Wonik Lee^1^, Sunwoo Chung^1^, Jisoo Kim^1,5^, Hyunjung Choi^1^, Hyeseung Song^4^, Chang Kon Lee^4^, Jae Hyun Jun^5^, Yong Jae Lee^5^, Kyunghyeon Lee^6^, Semi Kim^6^, Hye-ri Sim^6^, Young Il Choi^5^, Keun Ho Ryu^6^, Jong-Chan Park^1^, Dongjoon Lee^1^, Sun-Ho Han^1^, Daehee Hwang^2,7^, Jangbeen Kyung^5^, Inhee Mook-Jung^1,*^

^1^Department of Biochemistry and Biomedical Sciences, Seoul National University, College of Medicine, Seoul, Korea; ^2^Center for Plant Aging Research, Institute for Basic Science, DGIST, Daegu, Korea; ^3^Korea Brain Bank, Korea Brain Research Institute, Daegu, Korea; ^4^Department of Medicinal Chemistry, CKD Research Institute, CKD Pharmaceutical Company, Seoul, Korea; ^5^Department of Pharmacology, CKD Research Institute, CKD Pharmaceutical Company, Seoul, Korea; ^6^Department of Nonclinical Development, CKD Research Institute, CKD Pharmaceutical Company, Seoul, Korea; and ^7^Department of New Biology, DGIST, Daegu, Korea.

# These authors contributed equally.

J Yang and J Kim are currently present at CKD Research Institute

* Correspondence to : Tel: +82 2 740 8245; Fax: +82 2 3672 7352; E-mail: [inhee@snu.ac.kr](mailto:inhee@snu.ac.kr)

**SUPPORTING INFORMATION LISTING**

**Extended discussions**

**Supplemental Experimental Procedures**

**References**

**Supplementary Figures S1-8**

**Extended discussions**

We demonstrated that CKD-504 induced a greater interaction of tau with the Hsp40 family proteins, Dnaja1 and Dnaja2, and Hsp110 family protein, Hsph1. Members of the Hsp40 family regulate substrate specificity of Hsp70 and form a more stable complex with Hsp70 core chaperone and its substrates by stimulating the ATP hydrolysis activity of Hsp70(Sala *et al.* 2017). It has been reported that overexpression of Dnaja1 leads to clearance of tau via the UPS(Abisambra *et al.* 2012) and that Dnaja2 strongly inhibits tau aggregation(Mok *et al.* 2018). Members of the Hsp110 family act as disaggregases together with Hsp70 and Hsp40 family members(Shorter 2011) and play a role in maintaining tau in a normal phosphorylation state, which might contribute to blocking aggregation of tau(Eroglu *et al.* 2010). Based on these reports, CKD-504 may decrease tau aggregates by inhibiting hyperphosphorylation and aggregation or promoting disaggregation; it may also promote tau clearance by regulating the aforementioned chaperones and co-chaperones.

Other effects of HDAC6 inhibitors, such as rescue of Aβ-induced dysfunctions in mitochondrial axonal transport, excessive reactive oxygen species (ROS) production and elevated Ca^2+^, through increased acetylation of α-tubulin and peroxiredoxin 1 might also contribute to rescue of impaired memory (Kim *et al.* 2012; Choi *et al.* 2017). Indeed, the levels of acetylated α-tubulin were increased by CKD-504 in ADLP^APT^ mice in the preventive experimental paradigm (Figure S1f,g), and the Aβ-induced deficit in mitochondrial axonal transport, excessive ROS and Ca^2+^ were rescued by CKD-504 *in vitro* (Figure S8). A gene ontology analysis based on the differential tau interactome induced by CKD-504 also suggested that CKD-504 might contribute to stabilizing the cytoskeleton, which is involved in dendritic spine morphology and axonal transport, by increasing tau binding to proteins enriched in cytoskeleton organization (Figure S6).

As half-life of CKD-504 is short as 0.33 hour, we evaluated the duration of CKD-504 effects on mitochondria axonal transport after drug washout in cultures of Amyloid β (Aβ)-treated primary hippocampal neurons (Figure S8c). The defective mitochondrial movement in Aβ-treated neurons was rescued by CKD-504 and maintained up to 6 hours. This prolonged effect on mitochondria axonal transport may contribute to the *in vivo* efficacy of CKD-504 despite the short PK profile observed in animal studies.

**Supplemental Experimental Procedures**

**HDAC enzymatic assays**

The effect of CKD-504 (Chong Kun Dang, South Korea) on *in vitro* enzymatic activity of each HDAC enzyme isoform was performed by Reaction Biology Corporation (Malvern, USA). The enzyme assay is based on the unique fluorogenic substrate and developer combination as described previously(Wegener *et al.* 2003). The substrate for each HDAC isoform was as follows; fluorogenic peptide from p53 residues 379-382 (RHKK(Ac)AMC) for HDAC1, 2, 3, 6 and 10; fluorogenic HDAC Class2a substrate (Trifluoroacetyl Lysine) for HDAC4, 5, 7, 9 and 11; fluorogenic peptide from p53 residues 379-382 (RHK(Ac)K(Ac)AMC) for HDAC8. Briefly, HDAC enzyme was incubated in the assay buffer (50 mM Tris–HCl, pH 8.0, 137 mM NaCl, 2.7 mM KCl, 1 mM MgCl2, and 1 mg/mL bovine serum albumin). CKD-504 was delivered into the enzyme mixture then spun down and pre-incubated. The substrate was then added and the mixture was incubated to start the reaction for 1-2 h at 30°C. After the reaction was completed, a developer was added to stop the reaction and to generate fluorescent color. The fluorescence generated was read with excitation at 360 nM and emission at 460 nM by the EnVision Multilabel Plate Reader (PerkinElmer, Santa Clara, USA). The percentages of enzyme activity (relative to DMSO controls) and IC_50_ values were calculated using the GraphPad Prism 4 program based on a sigmoidal dose-response equation.

**Pharmacokinetics of CKD-504 in ADLP^APT^ mice**

For the PK profile of CKD-504, mouse blood and brain tissue including cerebrum, cerebellum and medulla oblongata were collected at the indicated time points after intraperitoneal injection of CKD-504. CKD-504 was extracted from plasma and brain tissue by protein precipitation using 90:10 (vol/vol) acetonitrile: methanol, and was analyzed using a LC-MS/MS method. The lower limit of quantification for all compounds was 0.5 ng/mL. PK parameters were estimated by noncompartmental model using WinNonlin (Pharsight, USA).

**Animals and intraperitoneal injections**

As described previously (Kim *et al.* 2018), ADLP^APT^ mice carry mutant human amyloid precursor protein (Swedish, Florida, and London), mutant human presenilin 1 (M146L and L286V), and mutant human tau (P301L). ADLP^APT^ mice carry mutant human amyloid precursor protein (Swedish, Florida, and London), mutant human presenilin 1 (M146L and L286V), and mutant human tau (P301L). Female wild type (WT) and ADLP^APT^ mice were used for brain tissue analysis after CKD-504 (Chong Kun Dang, South Korea) injection. We designed the preventive and the therapeutic models to determine the efficacy of CKD-504 before or after memory impairment which appeared around 6-month-old ADLP^APT^ mice(Kim *et al.* 2018). For the preventive model, CKD-504 (1 or 2.5 mg/kg) or saline were administered intraperitoneally to 4.5-month-old mice, twice a day, for four months. For the therapeutic model, 2.5 mg/kg of CKD-504 or saline were administered intraperitoneally to 6.5-month-old mice, twice a day, for two months. Animals were treated and maintained in accordance with the Animal Care and Use Guidelines of Seoul National University, Seoul, Korea.

**Behavioral tests**

For Y-maze test, a mouse was introduced to the middle of the maze and allowed to freely explore the maze for 8 min. Spontaneous alternation was measured to analyze spatial memory function. To calculate percentage of spontaneous alternation, the number of sequential entries to different Y-maze arms were divided by total arm entries. For contextual fear conditioning test (CFC), on day 1, a mouse was placed for a total of 5 min in an isolation cubicle with an electrifiable grid floor (Coulbourn, USA), with 0.55 mA foot shocks for 2 seconds each at 180 and 240 sec. On day 2, all of the experiments were performed in the same conditions as day 1, without a foot shock. A mouse was allowed to freely explore the same chamber for 3 min and the freezing behavior was analyzed by Freezeframe software (Coulbourn, USA).

**Golgi-Cox staining**

For Golgi-Cox staining, FD Rapid GolgiStain™ Kit (FD Neurnotechonogies, USA) was used. Mice were anesthetized by a mixture of Zoletil 50 (Virbac, France) and Rompun (3:1 ratio, 1 ml/kg, intramuscular injection). The brain was removed from the skull and quickly rinsed by double distilled water to remove blood. Brain tissue was immersed in the impregnation solution and stored at room temperature for 2 weeks in the dark. Brain tissue was transferred into solution C and stored at room temperature in the dark for 72 h. Tissue was sectioned using a cryostat and mounted on gelatin-coated microscope slides onto small drops of solution C. After drying at room temperature in the dark for 2 days, sections were stained by solution D and E, and then dehydrated and cleared by ethanol and xylene, respectively. Stained tissue was imaged by a confocal microscope (LSM700, Carl Zeiss). Secondary and tertiary dendrites of the cortical layers 3 and 5 pyramidal neurons were selected for analysis. Z-stacks of the dendrites up to 80 μm in depth were taken at 60x magnification with 0.5 μm intervals. Dendritic spines were measured manually using RECONSTRUCT software as described in the previous report (Risher *et al.* 2014).

**Immunohistochemistry**

Mice were anesthetized and perfused with phosphate-buffered saline (PBS). The brain tissue and human brain cortical organoids were soaked and fixed with 4% paraformaldehyde (PFA) solution in PBS for 24 h at 4 °C followed by incubation in 30% sucrose/PBS for 72 h at 4 °C. And then, they were preserved at -80 °C. The frozen brain tissue was sliced into 30 μm thick coronal sections using Leica CM 1850 Cryostat. The sliced samples were washed with PBS and then incubated in 70% formic acid/PBS for 20 min for retrieval of antigens to stain amyloid plaques or NFTs. After another washing with PBS, they were blocked with blocking solution (5% horse serum, 0.05% BSA, and 0.3% Triton X-100/PBS) for 1 h at room temperature. And then, they were incubated overnight with primary antibodies (4G8, covance, sig-39220; Tau-13, abcam, ab19030; AT8, Thermo Fisher scientific, MN1020; AT180, Thermo Fisher scientific, MN1040; Iba-1, Wako, 019-19741; MAP2, abcam, ab5392; Acetylated tubulin, Sigma aldrich, T7451; alpha tubulin, Millipore, 05-829)in the blocking solution. The brain slices were then labelled with fluorescent secondary antibodies (Alexa Fluor^TM^, Invitrogen, USA) against each corresponding primary antibody for 1 h at room temperature in the dark. The slices were imaged by LSM700 (Carl Zeiss) and quantified using Image J (NIH).

**Tau fractionation**

Tau fractionation was performed as previously described (Kim *et al.* 2018) with minor modification. In brief, one side of the hippocampus was homogenized in Tris-buffered saline (TBS) (25 mM Tris-HCl, pH 7.4, 150 mM NaCl, 1 mM EDTA and 1 mM EGTA with protease and phosphatase inhibitors). Homogenate was centrifuged at 14,000 g at 4 °C for 15 min. The supernatant was collected and quantified by BCA assay. And then, it was incubated with 1% N-lauroylsarcosine sodium salt solution (1% sarkosyl solution) (Sigma, USA) at 37°C for 1h. After that, it was ultra-centrifuged at 150,000 g for 45 min at 25 °C. The supernatant was collected for a sarkosyl-soluble fraction. The pellet was washed with 1% sarkosyl solution in TBS and ultra-centrifuged again at 150,000 g for 1 h at 25°C. The supernatant was removed and the pellet was collected for a sarkosyl-insoluble fraction. All fractions were boiled with sample buffer (Serva blue G) at 95 °C for 5 min.

**Cell culture and transfection**

HT22 and MEF cells were cultured in Dulbecco’s modified Eagle medium (DMEM; HyClone, USA) supplemented with 10% fetal bovine serum (Hyclone, USA) and 1% penicillin/streptomycin (Sigma, USA) at 37 °C in a 5% CO_2_ incubator. For transfection, DNA constructs were mixed with Lipofectamine LTX (Invitrogen, USA) in Opti-MEM (Gibco, USA) and the mixture was added to cells. HDAC6 KO mouse embryonic fibroblasts (MEFs) as well as WT MEFs cells were kindly provided by Prof. Dr. Lee, J. Y.

**DNA constructs and point mutations**

For tau, two tau constructs were used: pRK5-EGFP-Tau (Addgene, USA) and pRK5-EGFP-Tau P301L (Addgene, USA). For HDAC6, pCMV6-XL4-HDAC6 (Origene, USA) was used. For UBE2O, pcDNA3.1-3xFLAG-TEV-UBE2O (Addgene, USA). Those tau constructs were mutated using EZchange^TM^ (Enzynomics, Korea) according to manufacturer’s instructions. The kit was used to generate 4KQ (K274, 290, 321, 353Q) and 4KR (K274, 290, 321, 353R) tau mutations.

**Reagents**

CKD-504 (Chong Kun Dang, Korea), MG132 (Sigma, USA), Bafilomycin A1 (Sigma, USA), 3MA (Sigma, USA), leupeptin (Sigma, USA), NH_4_Cl (Sigma, USA), cycloheximide (Sigma, USA), BAPTA-AM (Thermo Fisher Scientific, USA), Fluo-4 (Thermo Fisher Scientific, USA), Trolox (Sigma, USA) and Aβ_1–42_ peptides (American peptide, USA and Bachem, Switzerland) were used for the experiments explained in the text.

**Immunoprecipitation and western blotting**

Cells and brain tissues were immunoprecipitated for western blotting and mass spectrometric analysis. Cells and tissue were lysed respectively in 1% Triton X-100 in TBS buffer (50 mM Tris HCl, 150 mM NaCl, pH 7.4) containing protease inhibitor and phosphatase inhibitor cocktails, phenyl-methylsulfonyl fluoride (PMSF) (Sigma, USA) and CKD-504 for HDAC6 inhibition. For immunoprecipitation of tau or GFP tagged tau, Tau-13 antibodies (Abcam, USA) or anti-GFP antibodies (Abcam, USA) were crosslinked to protein A/G agarose beads (SantaCruz, USA) by BS3 (Thermo Fisher Scientific, USA) according to manufacturer’s instructions, then incubated with lysates overnight at 4 °C. For western blot, precipitates were eluted with SDS-PAGE sample buffer by boiling at 95 °C for 3 min. For mass spectrometric analysis, brain tissue lysates precipitated with Tau-13 antibodies crosslinked to the beads were eluted by heating for 10 min at 50°C with 4% SDS elution buffer (4% SDS, 10 mM Tris-HCl (pH 7.4~7.8)). For the second elution, 4% SDS elution buffer was added again on the pelleted beads and the elution was performed under the same conditions. The two eluted samples were collected together and boiled for 5 min at 95°C for mass spectrometry. For immunoprecipitation of acetyl lysine (Ac-K), acetyl lysine affinity beads (Cytoskeleton, USA) were incubated with lysates overnight at 4 °C. Precipitates were eluted with SDS-PAGE sample buffer by boiling at 95 °C for 3 min. Total protein concentrations of cell and brain lysates were determined by the BCA assay. An equal amount of total protein extract was electrophoretically separated using sodium dodecyl sulphate polyacrylamide gel electrophoresis (SDS-PAGE) in 4–12% Bis–Tris gels transferred to polyvinylidene difluoride (PVDF) membranes or directly transferred to membrane for dot blotting. Blocked membranes (5% non-fat dry milk in TBS–0.1% Tween-20) were incubated with primary antibodies (PSD-95, abcam, ab18258; Tau-5, Thermo Fisher Scientific, AHB0042; Tau-13, abcam, ab19030; Tau[pT181], Thermo Fisher Scientific, 701530; Tau[pS199], Thermo Fisher Scientific, 44734G; Tau[pT231], Life Tech, 44746G; AT8, Thermo Fisher Scientific, MN1020; AT180, Thermo Fisher Scientific, MN1040; HDAC6, Cell signaling Technology, 7612s; Hsc70, Enzo Life Science, ADI-SPA-815; Hsp70, Cell signaling Technology, 4872; Rnf14, Abcam, ab134927; UBE2O, Bethyl, #A301-873A; Ubiquitin, Sigma, U5379; p-mTOR, Cell Signaling Technology, S2448; mTOR, Cell Signaling Technology, S2972; Beclin1, Abcam, ab62472; LC3B, Cell Signaling Technology, 2775; p62, Sigma, P0067; CatD, Santa Cruz, sc-6486; LAMP2, Abcam, ab13524; Acetylated lysine, Cell Signaling Technology, 9681; GFP, Abcam, ab1218) overnight at 4°C, and washed five times with TBS–0.1% Tween-20 (TBS-T) for 5 min. Membranes were then labelled with secondary IgG-HRP antibodies against each corresponding primary antibody. After washing with TBS-T, the membranes were incubated with ECL chemiluminescent reagent. Peroxidase activity was detected with LAS 4000 (GE healthcare life science, USA). The optical densities were normalized with a standard protein (β-actin, cell signaling technology, 3700S; α-tubulin, Millipore, 05-829)

**Aβ42 ELISA**

ELISA was performed as previously described (Song *et al.* 2015). ELISA was performed for quantifying Aβ42 and Aβ40 at the end of experiment on 8.5 month old ADLP^APT^ mice. Brain tissue was lysed in RIPA buffer and sonicated. Lysed brain tissue was ultracentrifuged at 100,000 g at 4°C for 1 hr. Supernatant was collected for RIPA soluble fraction. For RIPA insoluble fraction, the pellet was resuspended in 70% formic acid solution and ultracentrifuged at 100,000 g at 4°C for 1 hr. Protein concentrations were measured using a BCA assay. ELISA samples were run in duplicate on Aβ42 and Aβ40 ELISA following the protocol of the manufacturer (IBL, Japan). OD at 450 nm were read on a plate reader (Powerwave XS; BIO-TEK, Winooski, VT, USA).

**Generation of human brain cortical organoids from hiPSCs**

AD patient-derived hiPSCs (participant no.: CW50039) and healthy control-derived hiPSCs (participant no.: cw50071) were purchased from Coriell Institute for Medical Research (Camden, USA). Brain cortical organoids were generated using methods from the previous reports(Lin & Chen 2008; Seki *et al.* 2012; Pasca *et al.* 2015) with moderate modifications. Briefly, iPS Cells were plated onto a Matrigel (Corning, USA) -coated dish in the feeder-free maintenance medium for human ES and iPS Cells (mTeSR^TM^1, STEMCELL Technologies, USA). After incubation for 2-3 days, cells were transferred into hiPSC medium without FGF2: DMEM/F12 with GlutaMax (Thermo Fisher Scientific, USA) + 20% of Knock out serum (Thermo Fisher Scientific, USA) + 0.1 mM of MEM NEAA (Thermo Fisher Scientific, USA) + 100 U/ml penicillin (Sigma Aldrich Co., USA) + 100 μg/ml streptomycin (Sigma Aldrich Co., USA) + 0.1 mM of β-merchaptoethanol (Sigma-Aldrich Co., USA). The colonies were centrifuged and resuspended into the same hiPSC medium. To form embryoid bodies (EBs), suspended iPSC colonies were incubated for 48 hours and the aggregated EBs were moved to Corning low attachment plates. For the first five days, 10 μM of SB-431542 (Tocris, USA) and 10 μM of dorsomorphin (Sigma Aldrich Co., USA) were added, and then the floating spheroids were transferred into Neurobasal minus vitamin A medium (Thermo Fisher Scientific, USA) containing B27 Supplement minus vitamin A (Thermo Fisher Scientific), GlutaMAX^TM^ Supplement (Thermo Fisher Scientific, USA), 100 U/ml penicillin + 100 μg/ml streptomycin, 20 ng/ml of basic FGF2 (R&D system, USA), and EGF (EMD Millipore, USA). FGF2 and EGF were replaced with 100 μg/ml of BDNF (Peprotech, USA) and 100 μg/ml of NT3 (Peprotech, USA) on the twentieth day to promote neural differentiation, and the brain cortical organoids were then used for the experiments.

**Protein Digestion**

The samples were prepared from the following four conditions: Tau-13 IP samples from CKD-504 (Tau-CKD-504) or saline (Tau-saline) injected mice and IgG IP samples from CKD-504 (IgG-CKD-504) or saline (IgG-saline) injected mice. In each condition, we obtained three independent sets of brain samples from ADLP^APT^ mice, respectively (*n*=3; one mouse per sample). For 10-plex tandem mass tag (TMT) labeling, each pair of IgG IP control samples in IgG-CKD-504 and IgG-saline conditions were pooled to acquire the following 9 IP samples: three from Tau-CKD-504, Tau-saline, and IgG-CKD-504+IgG-salineFor each sample, the brain proteins were digested using the filter-aided sample preparation (FASP) method(Wisniewski *et al.* 2009) with slight modifications. Briefly, the proteins were reduced at 37°C with SDT buffer (4% SDS in 0.1M Tris-HCl pH 7.6 and 0.1 M DTT) for 45 min and then boiled at 95 °C for 10 min. Subsequently, protein samples were sonicated in bath sonicator (sonics, uibra cell, USA) for 10 min and centrifuged at 16,000 g for 5 min. The protein sample was transferred to a membrane filter device (YM-30, Millipore, USA) and mixed with 200 μL of 8 M urea in 0.1M Tris-HCl pH 8.5. The device was centrifuged at 14,000 g at 20 °C for 60 min to remove the SDS. This step was repeated for three times. Subsequently, proteins were alkylated with 100 μL of 50 mM iodoacetamide in 8 M urea for 25 min at room temperature in dark, followed by centrifugation at 14,000 g for 30 min. The filter was washed with 200 μL of 8 M urea for four times and then washed with 100 μL of 50 mM NH_4_HCO_3_ twice for buffer exchange. Trypsin (Promega, USA) was added to the proteins at an enzyme-to-protein ratio of 1: 50 (w/w), and the filter device was placed in a thermomixer (Eppendorf, Germany) and incubated at 37 °C for overnight. After the first digestion, the second digestion was carried out with additional trypsin (1:100 enzyme-to-protein ratio) at 37 °C for 6 hr. After digestion, the tryptic peptides were eluted by centrifugation at 14,000 g and 20 °C for 30 min. After collecting the tryptic peptides, the filter was rinsed with 60 μL of 50 mM NH_4_HCO_3_ and centrifuged at 14,000 g and 20 °C for 20 min, the eluent was combined with the first eluent. The combined eluent was dried by vacuum centrifugation, and the peptide concentration was determined by BCA assay. The peptide sample was divided into 10 μg units in Eppendorf tubes and kept at -80 °C until the subsequent TMT labeling.

**TMT Labeling and Peptide Fractionation**

The peptides from the aforementioned 9 IP samples were labeled with amine‐reactive TMT (TMT10 Label Reagents, #90111; Thermo Scientific, USA) according to the manufacturer's protocol (126, 128N, and 129C for Tau-saline; 127N, 128C, and 130N for Tau-CKD-504; and 127C, 129N, and 130C for IgG-saline+IgG-CKD-504). Each peptide sample was solubilized in 100 mM TEAB. The TMT labels were reconstituted in 41 μL of acetonitrile prior to the labeling, and 51 μL was added to each sample for labeling over 1 hr at 25 °C. Eight microliters of 5% hydroxylamine were added to quench the reaction in each sample. After 15 min, the 9 labeled peptide samples were combined. Finally, the combined peptide sample was dried using vacuum centrifugation. To increase the proteome coverage, mid-pH reverse phase liquid chromatography (RPLC) was performed at a flow rate of 0.5 mL/min using the 130 min gradient defined by solvent A (10mM TEAB in water, pH 7.4) and solvent B (10mM TEAB in 90 % ACN, pH 7.4). The gradient used is as follows: 0 % solvent B for 10 min, 0 - 5% solvent B in 5 min, 5 - 40 % in 85 min, 40 - 70 % in 5 min, 70 % for 10 min, 70 - 5 % in 10min, and 0% over 10 min. The 96 fractions were collected from 15 min to 110 min and were non-contiguously concatenated into 12 fractions by pooling two consecutive fractions from each of early (#1-24), first mid (#25-48), second mid (#49-72), and late (#73-96) sections of fractions. The 12 fractions were dried in a vacuum centrifuge concentrator and stored at –80°C until LC-MS/MS experiments.

**LC–MS/MS Analysis**

TMT‐labeled peptides (1 μg) from each of 12 fractions were dissolved in solvent A (2% acetonitrile and 0.1% formic acid). Nano‐LC‐MS/MS analyses were performed using a Q Exactive Mass Spectrometer (Thermo Scientific, USA) equipped with an EASY‐Spray Ion Source and coupled to an EASY‐nLC 1000 (Thermo Scientific, USA). Peptides were loaded onto an Acclaim PepMap 100 pre‐column (75 μm × 2 cm, C18, 3‐μm particles, 100 Å pore size) and separated on an ES800 Easy‐Spray column (50 cm × 75 μm inner diameter, PepMap C18, 3‐μm particles, 100 Å pore size). A 180‐min gradient was used at a flow rate of 300 nL/min: from 2 to 40% solvent B (98% acetonitrile and 0.1% formic acid) over 120 min, from 40 to 80% solvent B over 30 min, 80% solvent B for 15 min, and 2% solvent B for 15 min. The temperature of the column was maintained at 35 °C, and the electrospray voltage was set to be 1.7 kV. MS precursor scans (m/z range of 450–2000 Th) were acquired with the following setting: an automated gain control (AGC) target value = 3.0 × 10^6^, resolution = 70,000, and maximum ion injection time = 250 ms. The MS/MS data for up to the ten most abundant ions were acquired in a data‐dependent mode using higher energy collisional dissociation with the following setting: normalized collision energy = 32, resolution = 35,000, AGC target value = 2.0 × 10^5^, and maximum injection time = 120 ms.

**LC–MS/MS Data Analysis**

For each MS/MS dataset, post experiment monoisotopic mass refinement (PE‐MMR) was used to accurately assign precursor mass to the MS/MS data(Shin *et al.* 2008). The resulting MS/MS data (i.e., mgf files) were subjected to a database search using the MS‐GF+ search engine (v2017.01.13)(Kim & Pevzner 2014) against the UniProt‐Prot‐Mouse‐reference database (released February, 2016; 58,970 entries) with the following parameters: precursor mass tolerance = 10 ppm; non-tryptic, static modifications of carbamidomethylation (+57.021460 Da) to cysteine; and TMT (+229.162932 Da) to N‐termini and lysine and variable modifications of oxidation (+15.994920 Da) to methionine; carbamylation (+43.005810 Da) to N-termini; acetylation (+42.010565 Da) to lysine; phosphorylation (+79.966331 Da) to Serine, threonine, and tyrosine; and ubiquitination (+114.042927 Da) to lysine. The search results from the 12 MS/MS datasets were combined. The peptide spectrum matches (PSMs) were obtained using the false discovery rate (FDR) of 1%.

**Identification of Differentially Interacting Proteins (DIPs)**

The reporter ion intensities of the identified peptides were converted to log_2_-intensities and then normalized using the quantile-normalization method (Bolstad *et al.* 2003). We developed a non-linear model that matches the intensity distributions of tau peptides from Tau-saline and Tau-CKD-504, and the intensities of non-tau peptides from Tau-CKD-504 were then adjusted to the one that would be observed in Tau-saline using the non-linear model. Specifically, 1) we sorted tau peptide intensities from Tau-saline and Tau-CKD-504 in the ascending order and developed a non-linear model that matches the intensities from Tau-CKD-504 to those from Tau-saline at the same quantile (rank); 2) and for each of non-tau peptides from Tau-CKD-504, we estimated a quantile for the non-tau peptide intensity in the intensity distribution of tau peptides from Tau-CKD-504 using the linear interpolation; and 3) using the non-linear model, we calculated a tau peptide intensity from Tau-saline for the estimated quantile and then adjusted the peptide intensity from Tau-CKD-504 to the calculated tau peptide intensity. Using the normalized intensities, we identified differentially interacting peptides in the two comparisons (Tau-CKD-504 versus Tau-saline and Tau-CKD-504 versus IgG-CKD-504+IgG-saline) using the statistical method previously described(Bolstad *et al.* 2003). For each comparison, two-tailed Student’s t-test (e.g., 3 samples in Tau-CKD-504 versus 3 samples in Tau-saline) was applied to calculate T-values for the non-tau peptides. To compute P-values for these T-values, we estimated an empirical distribution of T-values for the null hypothesis (i.e., a peptide is not differentially interacted) by performing all possible random permutations of the samples and then by applying the Gaussian kernel density estimation method to T-values resulted from the random permutations(Bowman & Azzalini 1997). For each peptide, the FDRs were computed by the two-sided test using the empirical null distribution using Storey’s method(Storey & Tibshirani 2003). The differentially interacted peptides were selected as the ones with FDR ≤ 0.05 and absolute log_2_-fold-changes ≥ 0.58 (1.5-fold) in each comparison. To identify the peptides for the proteins with increased interactions in Tau-CKD-504, compared with in Tau-saline, among the selected differentially interacted peptides, we further selected the ones that were up-regulated in Tau-CKD-504, compared with both in IgG-CKD-504+IgG-saline and Tau-saline. Finally, we selected a set of for the proteins with increased interactions in Tau-CKD-504 as the proteins that have more than two selected differentially interacted peptides. Moreover, to understand cellular processes associated with these interactors, functional enrichment analysis was performed using DAVID software to identify GO biological processes (GOBPs) represented by the interactors(Huang *et al.* 2009). The GOBPs enriched by the DIPs were identified as the ones with P ≤ 0.05.

**Mitochondrial transport imaging**

Details of microfluidic chambers and analysis of axonal transport of mitochondria were described in the reference (Kim *et al.* 2012). In brief, rat primary neurons cultured in microfluidic chambers were transfected with pDsRed2-Mito at 7 days *in vitro* (DIV) to observe mitochondria. At 8 DIV, the neurons were treated with 2 μM of Aβ_1-42_ peptides and 21 h after Aβ_1-42_ peptides treatment, CKD-504 was added for 3 h.

**DCFDA assay (ROS measurement)**

For measuring ROS, cells were treated with 1 μM of cell-permeant 2′, 7′-dichlorodihydrofluorescein diacetate (DCFDA, Invitrogen, USA). After 1 h incubation at 37°C, DMEM containing DCFDA was changed with fresh DMEM. Using a fluorescence microscope (Olympus, Japan) or CellInsight (Thermo Fisher scientific, USA), fluorescent signals were captured then analyzed with Image J (NIH) or CellInsight software.

**Fluo-4 assay (Ca^2+^ measurement)**

For Ca^2+^ concentration measurement, cells were incubated with Fluo-4 (Invitrogen, USA) at 37°C. After 1 h, the medium was changed with fresh one. Florescent signals were captured by CellInsight or a fluorescence microscope. Images were analyzed by Image J (NIH) or CellInsight software.

**Statistical analysis**

All data were analyzed by two-way analysis of variance (ANOVA) or one-way ANOVA with Bonferroni post-hoc tests or by analysis of covariance (ANCOVA) or Student’s *t*-tests. All data were shown as mean ± SEM.

**References**

Abisambra JF, Jinwal UK, Suntharalingam A, Arulselvam K, Brady S, Cockman M, Jin Y, Zhang B, Dickey CA (2012). DnaJA1 antagonizes constitutive Hsp70-mediated stabilization of tau. *J Mol Biol*. **421**, 653-661.

Bolstad BM, Irizarry RA, Astrand M, Speed TP (2003). A comparison of normalization methods for high density oligonucleotide array data based on variance and bias. *Bioinformatics*. **19**, 185-193.

Bowman AW, Azzalini A (1997). *Applied smoothing techniques for data analysis : the kernel approach with S-Plus illustrations*. Oxford

New York: Clarendon Press ;

Oxford University Press.

Choi H, Kim HJ, Kim J, Kim S, Yang J, Lee W, Park Y, Hyeon SJ, Lee DS, Ryu H, Chung J, Mook-Jung I (2017). Increased acetylation of Peroxiredoxin1 by HDAC6 inhibition leads to recovery of Abeta-induced impaired axonal transport. *Mol Neurodegener*. **12**, 23.

Eroglu B, Moskophidis D, Mivechi NF (2010). Loss of Hsp110 leads to age-dependent tau hyperphosphorylation and early accumulation of insoluble amyloid beta. *Mol Cell Biol*. **30**, 4626-4643.

Huang DW, Sherman BT, Lempicki RA (2009). Systematic and integrative analysis of large gene lists using DAVID bioinformatics resources. *Nature Protocols*. **4**, 44-57.

Iqbal K, Liu F, Gong CX (2016). Tau and neurodegenerative disease: the story so far. *Nat Rev Neurol*. **12**, 15-27.

Ittner LM, Ke YD, Delerue F, Bi M, Gladbach A, van Eersel J, Wolfing H, Chieng BC, Christie MJ, Napier IA, Eckert A, Staufenbiel M, Hardeman E, Gotz J (2010). Dendritic function of tau mediates amyloid-beta toxicity in Alzheimer's disease mouse models. *Cell*. **142**, 387-397.

Jinwal UK, Miyata Y, Koren J, 3rd, Jones JR, Trotter JH, Chang L, O'Leary J, Morgan D, Lee DC, Shults CL, Rousaki A, Weeber EJ, Zuiderweg ER, Gestwicki JE, Dickey CA (2009). Chemical manipulation of hsp70 ATPase activity regulates tau stability. *J Neurosci*. **29**, 12079-12088.

Kim C, Choi H, Jung ES, Lee W, Oh S, Jeon NL, Mook-Jung I (2012). HDAC6 inhibitor blocks amyloid beta-induced impairment of mitochondrial transport in hippocampal neurons. *PLoS One*. **7**, e42983.

Kim DK, Park J, Han D, Yang J, Kim A, Woo J, Kim Y, Mook-Jung I (2018). Molecular and functional signatures in a novel Alzheimer's disease mouse model assessed by quantitative proteomics. *Mol Neurodegener*. **13**, 2.

Kim S, Pevzner PA (2014). MS-GF+ makes progress towards a universal database search tool for proteomics. *Nat Commun*. **5**, 5277.

Lin Y, Chen G (2008). Embryoid body formation from human pluripotent stem cells in chemically defined E8 media. In *StemBook*). Cambridge (MA).

Mayer MP (2013). Hsp70 chaperone dynamics and molecular mechanism. *Trends Biochem Sci*. **38**, 507-514.

Mok SA, Condello C, Freilich R, Gillies A, Arhar T, Oroz J, Kadavath H, Julien O, Assimon VA, Rauch JN, Dunyak BM, Lee J, Tsai FTF, Wilson MR, Zweckstetter M, Dickey CA, Gestwicki JE (2018). Mapping interactions with the chaperone network reveals factors that protect against tau aggregation. *Nat Struct Mol Biol*. **25**, 384-393.

Pasca AM, Sloan SA, Clarke LE, Tian Y, Makinson CD, Huber N, Kim CH, Park JY, O'Rourke NA, Nguyen KD, Smith SJ, Huguenard JR, Geschwind DH, Barres BA, Pasca SP (2015). Functional cortical neurons and astrocytes from human pluripotent stem cells in 3D culture. *Nat Methods*. **12**, 671-678.

Pooler AM, Noble W, Hanger DP (2014). A role for tau at the synapse in Alzheimer's disease pathogenesis. *Neuropharmacology*. **76 Pt A**, 1-8.

Risher WC, Ustunkaya T, Singh Alvarado J, Eroglu C (2014). Rapid Golgi analysis method for efficient and unbiased classification of dendritic spines. *PLoS One*. **9**, e107591.

Roberson ED, Scearce-Levie K, Palop JJ, Yan F, Cheng IH, Wu T, Gerstein H, Yu GQ, Mucke L (2007). Reducing endogenous tau ameliorates amyloid beta-induced deficits in an Alzheimer's disease mouse model. *Science*. **316**, 750-754.

Sala AJ, Bott LC, Morimoto RI (2017). Shaping proteostasis at the cellular, tissue, and organismal level. *J Cell Biol*. **216**, 1231-1241.

Seki T, Yuasa S, Fukuda K (2012). Generation of induced pluripotent stem cells from a small amount of human peripheral blood using a combination of activated T cells and Sendai virus. *Nat Protoc*. **7**, 718-728.

Shin B, Jung HJ, Hyung SW, Kim H, Lee D, Lee C, Yu MH, Lee SW (2008). Postexperiment monoisotopic mass filtering and refinement (PE-MMR) of tandem mass spectrometric data increases accuracy of peptide identification in LC/MS/MS. *Mol Cell Proteomics*. **7**, 1124-1134.

Shorter J (2011). The mammalian disaggregase machinery: Hsp110 synergizes with Hsp70 and Hsp40 to catalyze protein disaggregation and reactivation in a cell-free system. *PLoS One*. **6**, e26319.

Song H, Chang YJ, Moon M, Park SK, Tran PT, Hoang VH, Lee J, Mook-Jung I (2015). Inhibition of glutaminyl cyclase ameliorates amyloid pathology in an animal model of Alzheimer's disease via the modulation of gamma-secretase activity. *J Alzheimers Dis*. **43**, 797-807.

Storey JD, Tibshirani R (2003). Statistical significance for genomewide studies. *Proc Natl Acad Sci U S A*. **100**, 9440-9445.

Suberbielle E, Sanchez PE, Kravitz AV, Wang X, Ho K, Eilertson K, Devidze N, Kreitzer AC, Mucke L (2013). Physiologic brain activity causes DNA double-strand breaks in neurons, with exacerbation by amyloid-beta. *Nat Neurosci*. **16**, 613-621.

Vossel KA, Zhang K, Brodbeck J, Daub AC, Sharma P, Finkbeiner S, Cui B, Mucke L (2010). Tau reduction prevents Abeta-induced defects in axonal transport. *Science*. **330**, 198.

Wang Y, Mandelkow E (2016). Tau in physiology and pathology. *Nat Rev Neurosci*. **17**, 5-21.

Wegener D, Wirsching F, Riester D, Schwienhorst A (2003). A fluorogenic histone deacetylase assay well suited for high-throughput activity screening. *Chem Biol*. **10**, 61-68.

Wisniewski JR, Zougman A, Nagaraj N, Mann M (2009). Universal sample preparation method for proteome analysis. *Nat Methods*. **6**, 359-362.

Young ZT, Rauch JN, Assimon VA, Jinwal UK, Ahn M, Li X, Dunyak BM, Ahmad A, Carlson GA, Srinivasan SR, Zuiderweg ER, Dickey CA, Gestwicki JE (2016). Stabilizing the Hsp70-Tau Complex Promotes Turnover in Models of Tauopathy. *Cell Chem Biol*. **23**, 992-1001.

Zhang L, Liu C, Wu J, Tao JJ, Sui XL, Yao ZG, Xu YF, Huang L, Zhu H, Sheng SL, Qin C (2014). Tubastatin A/ACY-1215 improves cognition in Alzheimer's disease transgenic mice. *J Alzheimers Dis*. **41**, 1193-1205.

Zhou L, McInnes J, Wierda K, Holt M, Herrmann AG, Jackson RJ, Wang YC, Swerts J, Beyens J, Miskiewicz K, Vilain S, Dewachter I, Moechars D, De Strooper B, Spires-Jones TL, De Wit J, Verstreken P (2017). Tau association with synaptic vesicles causes presynaptic dysfunction. *Nat Commun*. **8**, 15295.

**Supplementary Figures**

**
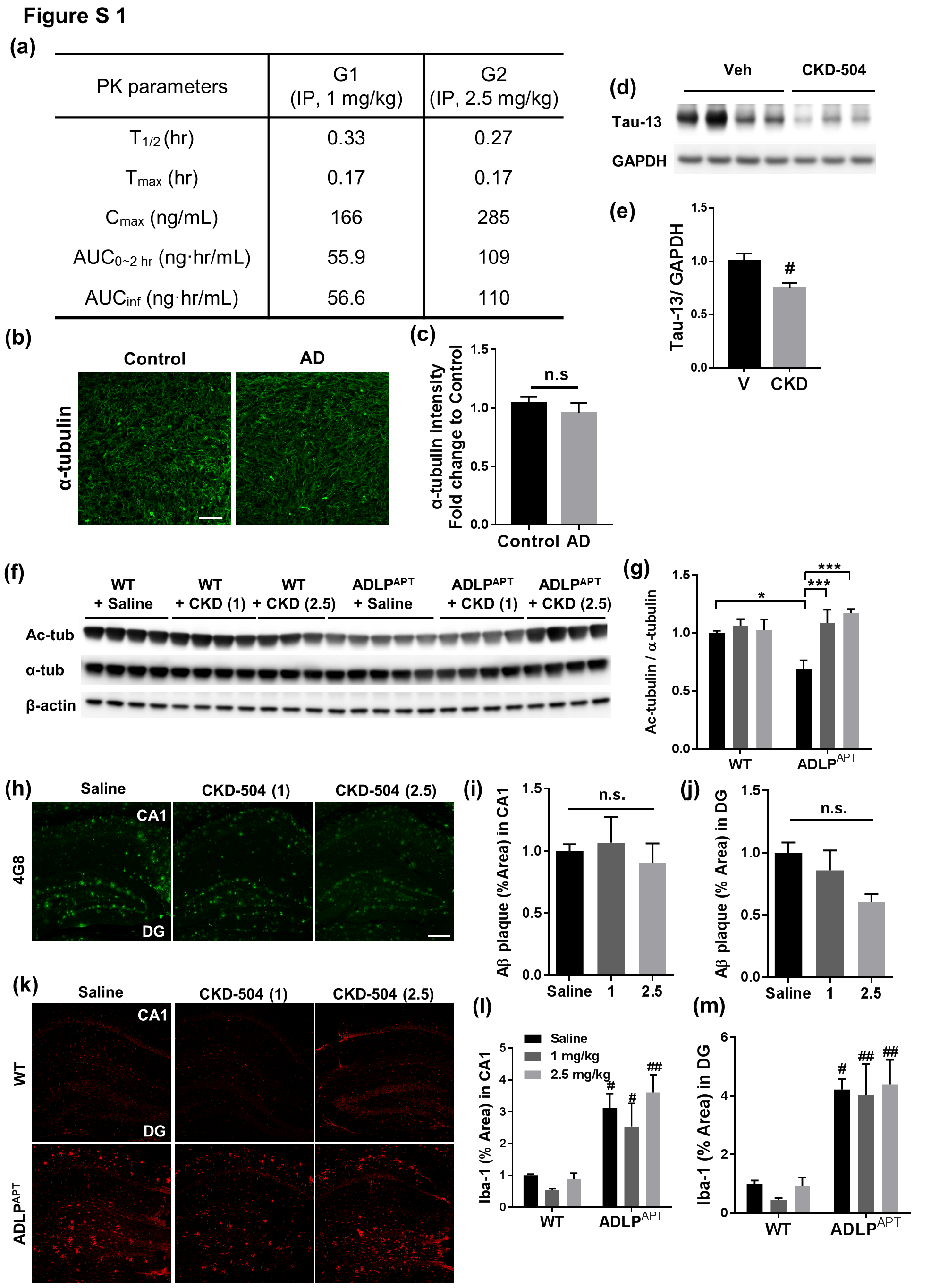
**

**Figure S1.** **CKD-504 did not alter Aβ plaques and neuroinflammation in the preventive model of ADLP^APT^ mice.**

(a) Pharmacokinetics of CKD-504. (b),(c) α-tubulin shows no differences between healthy control and AD patient-derived brain organoids. Representative images (b) and quantification (c). Data are presented as means ± SEM. Student’s *t*-test. n.s : non significant. Scale bar: 50 μm.(d),(e) Effect of CKD-504 on healthy control brain organoids. Representative images (d) and quantification (e). Data are presented as means ± SEM. Student’s *t*-test. *#P* < 0.05. (f),(g) CKD-504 recovered the level of acetylated α-tubulin in ADLP^APT^ mice. Representative images (f) and quantification (g). Data are presented as means ± SEM. two-way ANOVA followed by Bonferroni post-hoc test. **P* < 0.05, ****P* < 0.01. (h-j) Aβ plaques were not changed by CKD-504 in ADLP^APT^ mice. Representative images of brain slices stained by anti-Aβ antibody (4G8) in the hippocampus (h) and quantification (i,j) (n=4-8). Data are presented as means ± SEM. One-way ANOVA followed by Bonferroni post-hoc test. n.s.: non-significant. (k-m) Neuroinflammation was not changed by CKD-504 in ADLP^APT^ mice. Representative images of brain slices stained by anti-Iba-1 antibody in the hippocampus (k) and quantification (l,m) (n=4-6). Scale bar: 100 μm. Data are presented as means ± SEM. Student’s *t* -tests compared with WT. *#P* < 0.05, *##P* < 0.01.


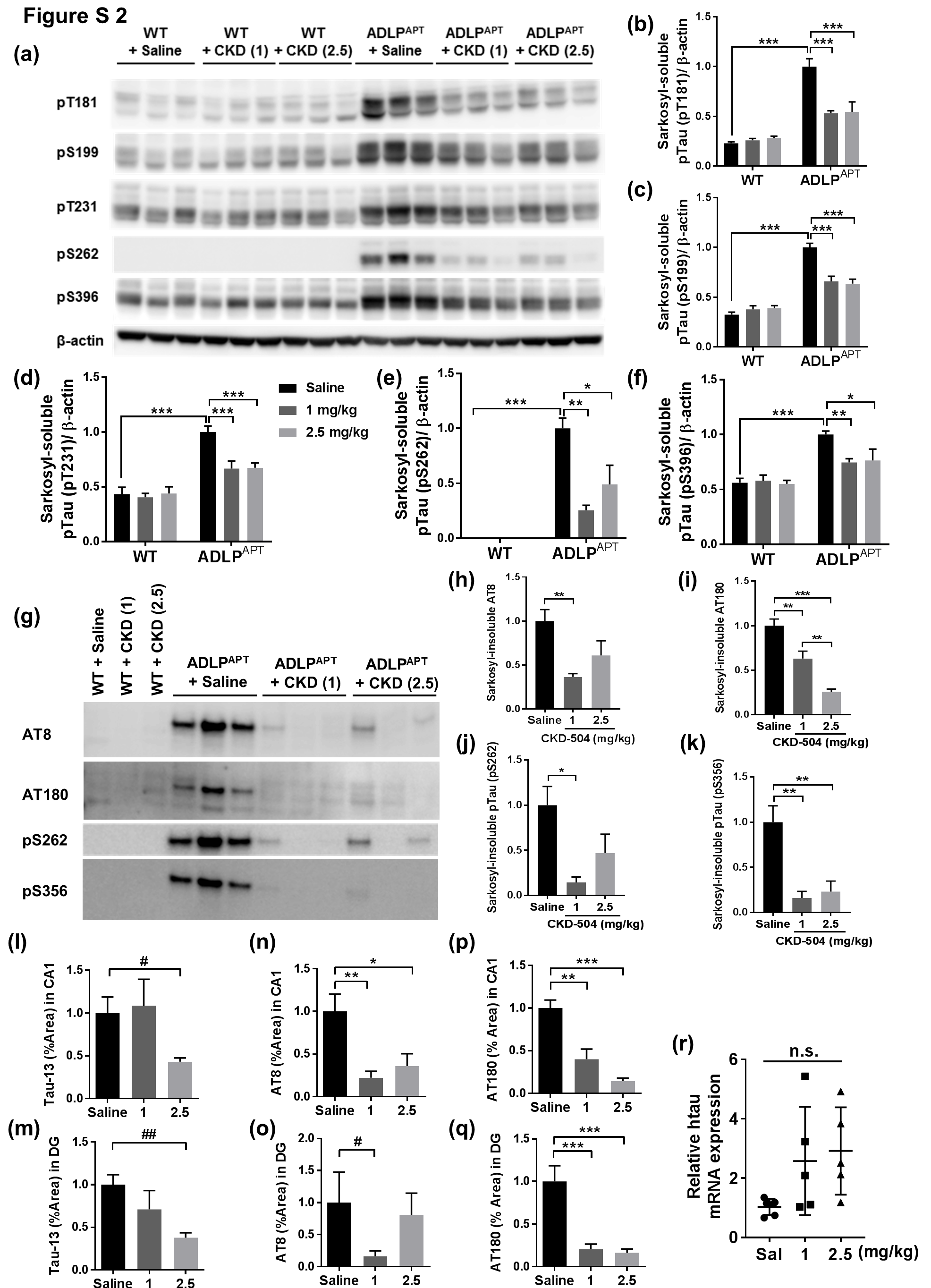


**Figure S2.** **CKD-504 reduced sarkosyl-soluble and –insoluble, phosphorylated tau in the preventive model of ADLP^APT^ mice (Related to Figure 2).**

(a-f) Sarkosyl-soluble, phosphorylated tau was reduced by CKD-504. Quantification of sarkosyl-soluble, phosphorylated tau (n=5-6). Data are presented as means ± SEM. Two-way ANOVA followed by Bonferroni post-hoc test. **P* < 0.05, ***P* < 0.01, ****P* < 0.001. (g-k) Sarkosyl-insoluble, phosphorylated tau was reduced by CKD-504. Quantification of sarkosyl- insoluble, phosphorylated tau (n=5-6). (h-q) Quantification of total and phosphorylated tau proteins in the hippocampus as confirmed by immunohistochemistry (Related to Figure 2j). (r) hTau mRNA expression was not changed by CKD-504. Data are presented as means ± SEM. One-way ANOVA followed by Bonferroni post-hoc test. n.s : non-significant **P* < 0.05, ***P* < 0.01, ****P* < 0.001.

**
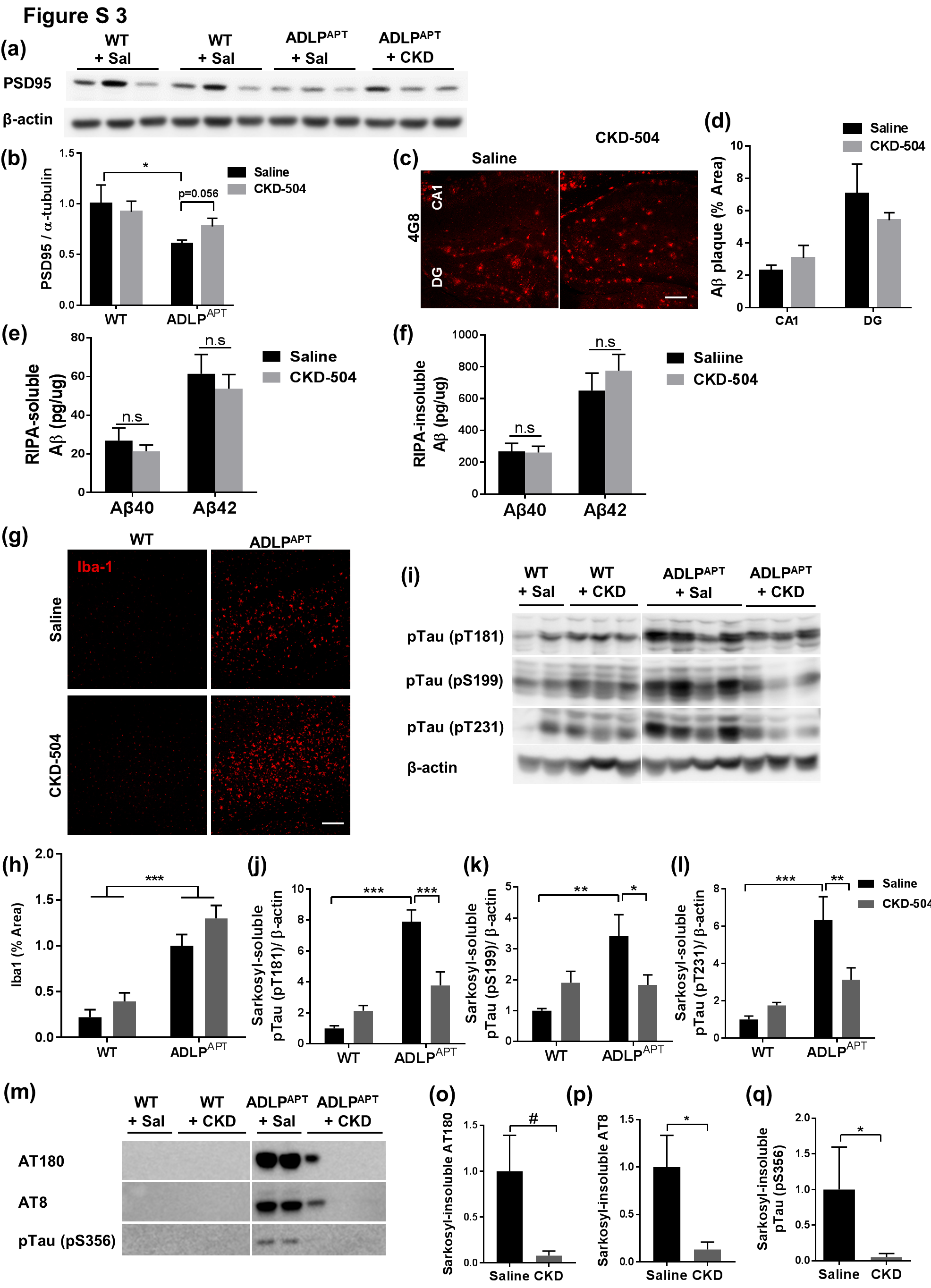
**

**Figure S3.** **CKD-504 did not alter Aβ plaques and neuroinflammation, but reduced phosphorylated tau in the** **therapeutic model of ADLP^APT^ mice (Related to Figure 3).**

(a),(b) PSD95 level showed recovering tendency by CKD-504 in ADLP^APT^ mice. Representative images (a) and quantification (b). (c),(d) Aβ plaques were not changed by CKD-504 in ADLP^APT^ mice. Representative images of brain slices stained by anti-Aβ antibody (4G8) in the hippocampus (c) and quantification (d) (n=3-6). (e),(f) RIPA-soluble and -insoluble Aβ was analyzed by ELISA and no significant changes were shown by CKD-504. Quantification of RIPA-soluble Aβ (e) and RIPA-insoluble Aβ (f). (g),(h) Neuroinflammation was not changed by CKD-504 in ADLP^APT^ mice. Representative images of brain slices stained by anti-Iba-1 antibody in the hippocampus (g) and quantification (h) (n=5-10). (i-q) Sarkosyl-soluble and –insoluble, phosphorylated tau were reduced by CKD-504. Representative images (i,m) and quantification (j-l, n-p) (n=3-4). Data are presented as means ± SEM. Student’s *t* –tests (d,e,f,o-q). Two-way ANOVA followed by Bonferroni post-hoc test (b,h,j,k,l). **P* < 0.05, ***P* < 0.01, ****P* < 0.001.

**
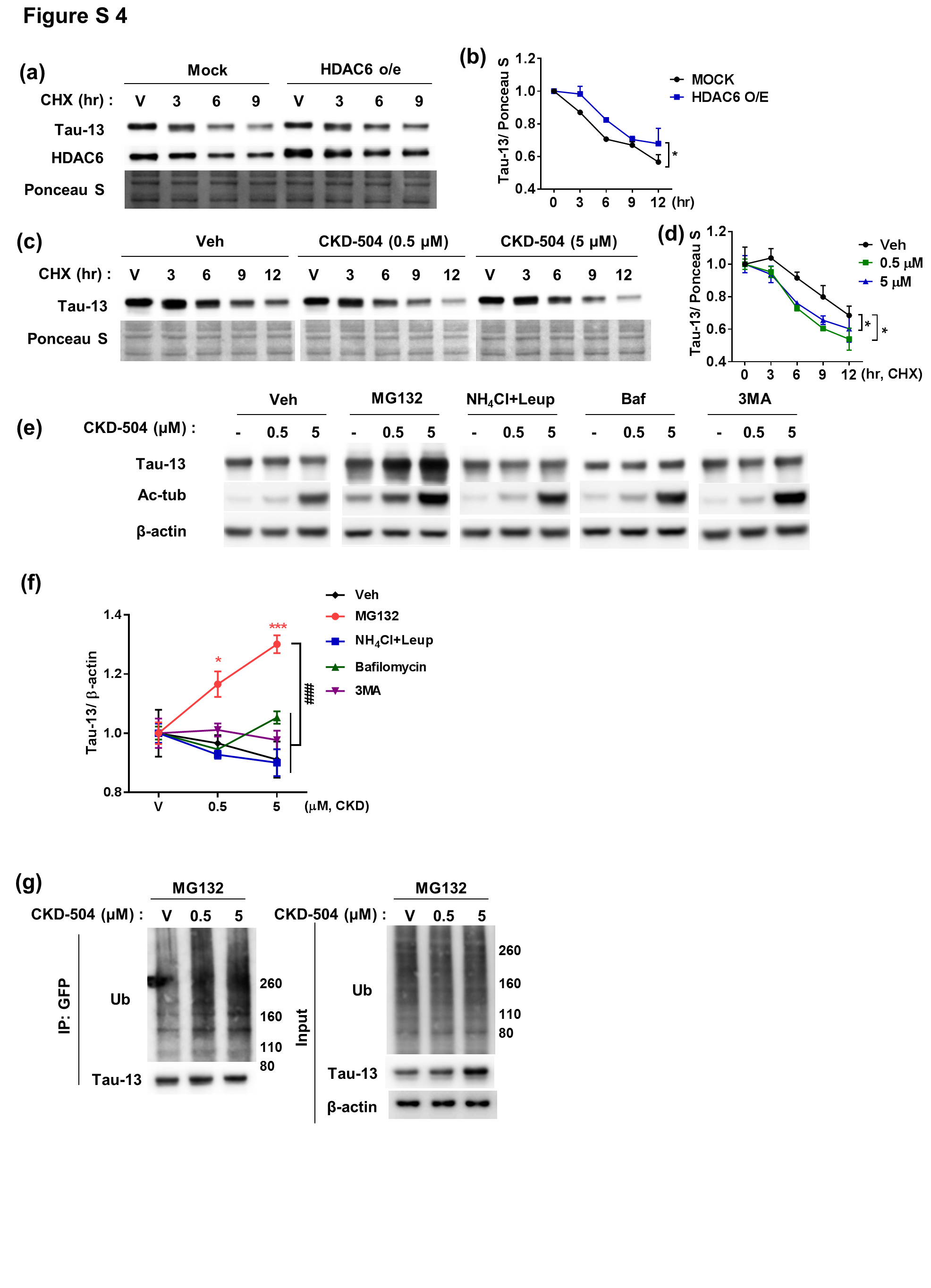
**

**Figure S4.** **CKD-504 potentiates proteasomal degradation of tau in HT22 cells.**

(a), (b) HDAC6 overexpression reduced degradation rate of tau in HT22 cells. Representative images (a) and quantification (b) (n=3, independent experiments). (c), (d) Tau degradation rate was accelerated by CKD-504 in HT22 cells. HT22 cells were treated with CKD-504 for 12 h. Representative images (c) and quantification (d) (n=3, independent experiments). Cycloheximide (50 μg/ml) was treated for the indicated times (a-d). Data are presented as means ± SEM. Analysis of covariance (ANCOVA). *P<0.05, ****P* < 0.001. (e), (f) Tau was accumulated in CKD-504 and MG132 (5 μM) co-treated HT22 cells, but not in vehicle, NH_4_Cl (20 mM), leupeptin (0.1 mM), bafilomycin (10 nM) or 3MA (5 mM) treated HT22 cells. Representative images (e) and quantification (f) (n=5, independent experiments). Data are presented as means ± SEM. one-way ANOVA followed by Bonferroni post-hoc test within MG132 treated group. ***P* < 0.01, two-way ANOVA followed by Bonferroni post-hoc test among vehicle and inhibitor treated groups. *###P* < 0.001. (g) Ubiquitination of tau was increased by CKD-504 in HT22 cells. Human tau was immunoprecipitated by anti-GFP antibody. CHX : cycloheximide, Veh : Vehicle, Baf: bafilomycin, Leup : leupeptin, 3MA : 3-Methyladenine, Ub: ubiquitin.


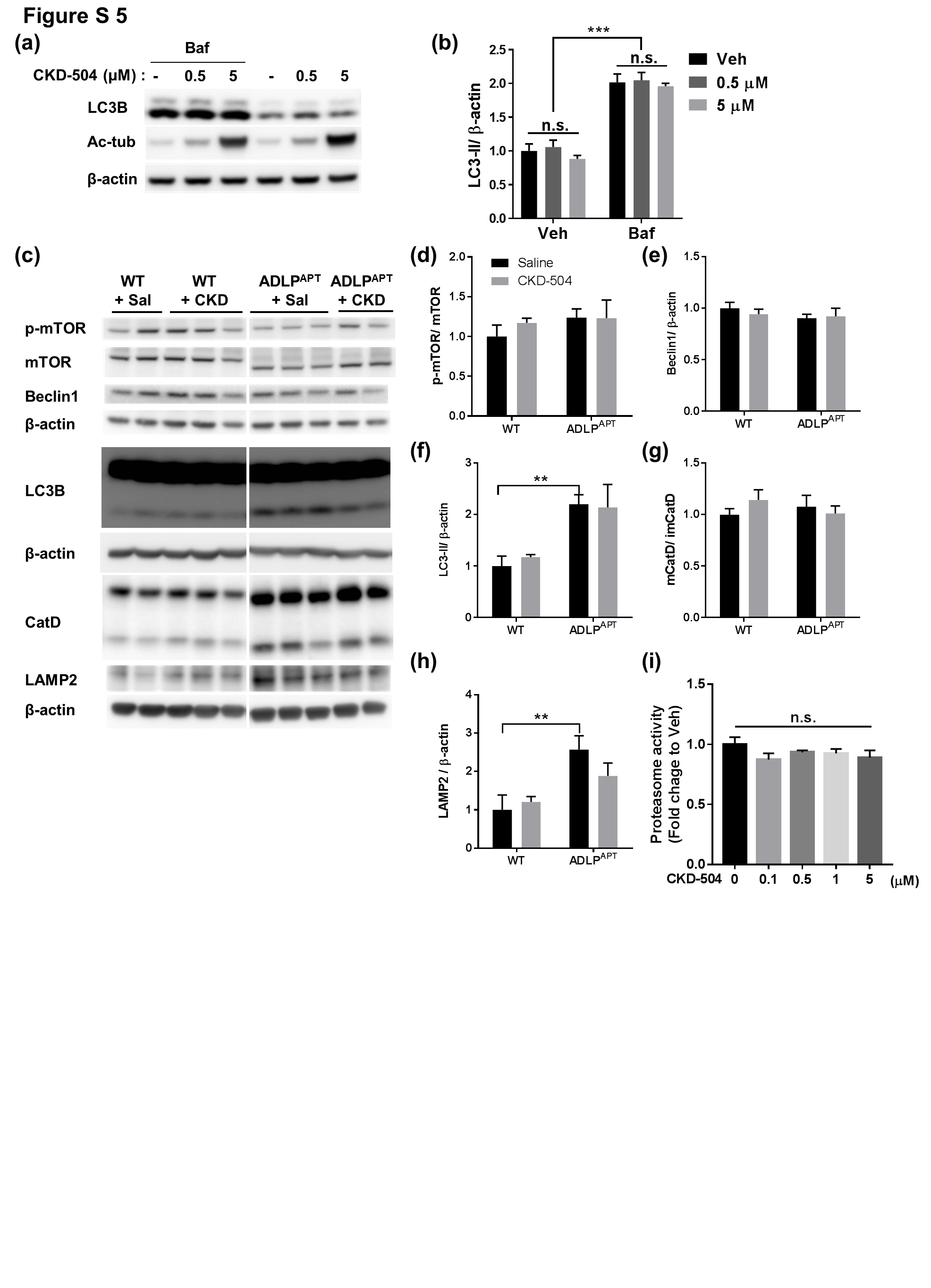


**Figure S5 Autophagic flux was not altered by CKD-504.**

(a),(b) HT22 cells were treated with CKD-504 in the presence or absence of bafilomycin. Representative images (a) and quantification (b). (n=4, independent experiments). Data are presented as means ± SEM. one-way ANOVA followed by Bonferroni post-hoc test within each treatment group. n.s.: non-significant., two-way ANOVA followed by Bonferroni post-hoc test between vehicle and bafilomycin treated groups. ****P* < 0.001. (c-h) ALS-related proteins were not changed by CKD-504. Cortical lysates were immunoblotted and detected with the indicated antibodies. Representative immunoblot images (c) and quantification of protein levels (d-h) (n=5-8). two-way ANOVA followed by Bonferroni post-hoc test. ***P* < 0.01 (i) Proteasome activity was not changed by CKD-504. HT22 cells were treated with CKD-504 with the indicated concentrations. Quantification of protease activity (i) (n=4, independent experiments). One-way ANOVA followed by Bonferroni post-hoc test. n.s. : non-significant.

**
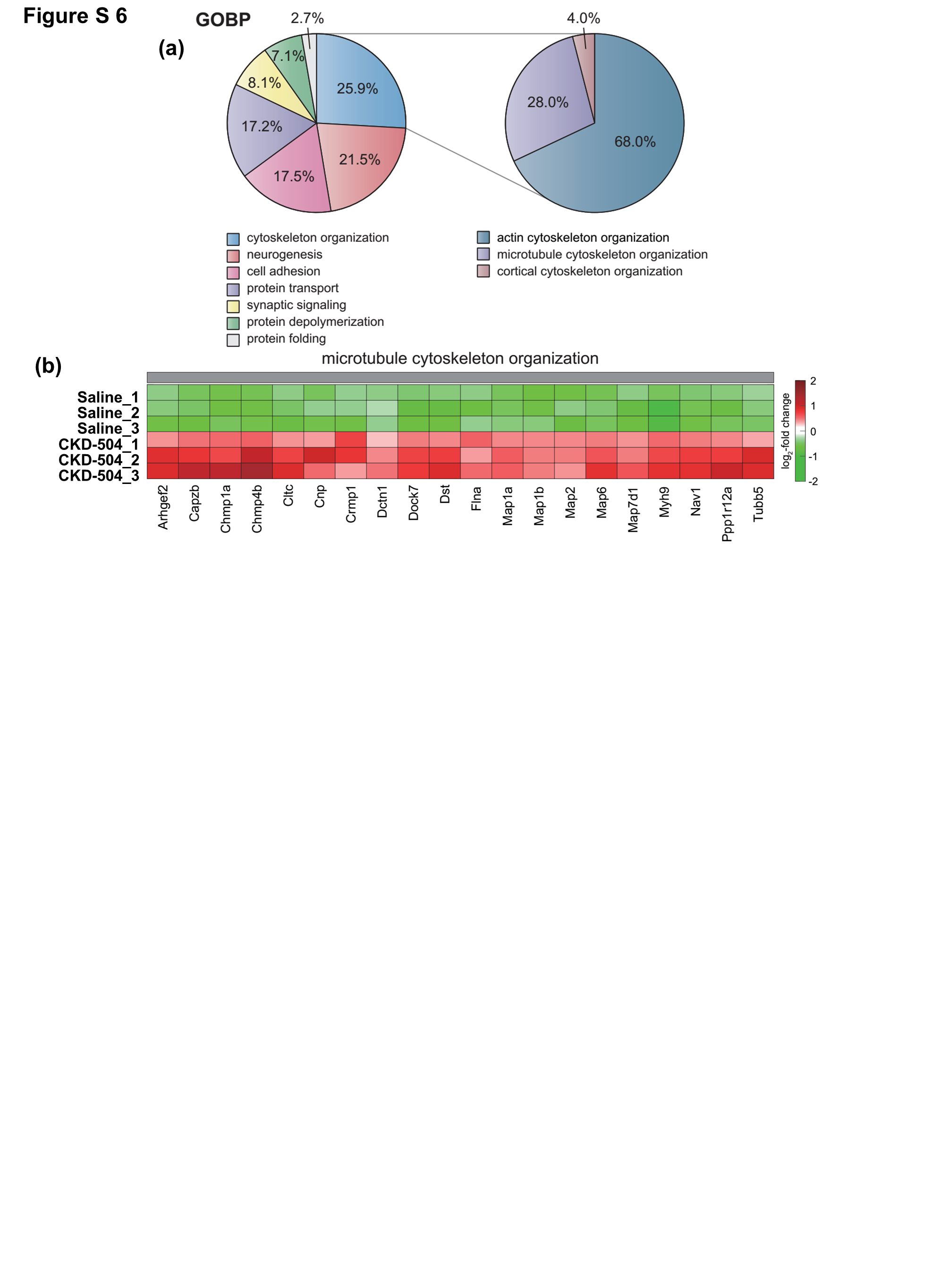
**

**Figure S6.** **Tau interactome was changed by CKD-504 in ADLP^APT^ mice.**

(a) Major GOBPs of tau interactors whose interaction strength was altered by CKD-504 in ADLP^APT^ mice are shown. (b) CKD-504 increased interactions of tau with proteins associated with microtubule cytoskeleton organization in ADLP^APT^ mice. The heat map represents log_2_-fold changes of intensities reflecting interaction strength, which were measured by mass spectrometric analysis in CKD-504-treated samples relative to saline controls. The color bar shows a gradient of log_2_-fold changes.


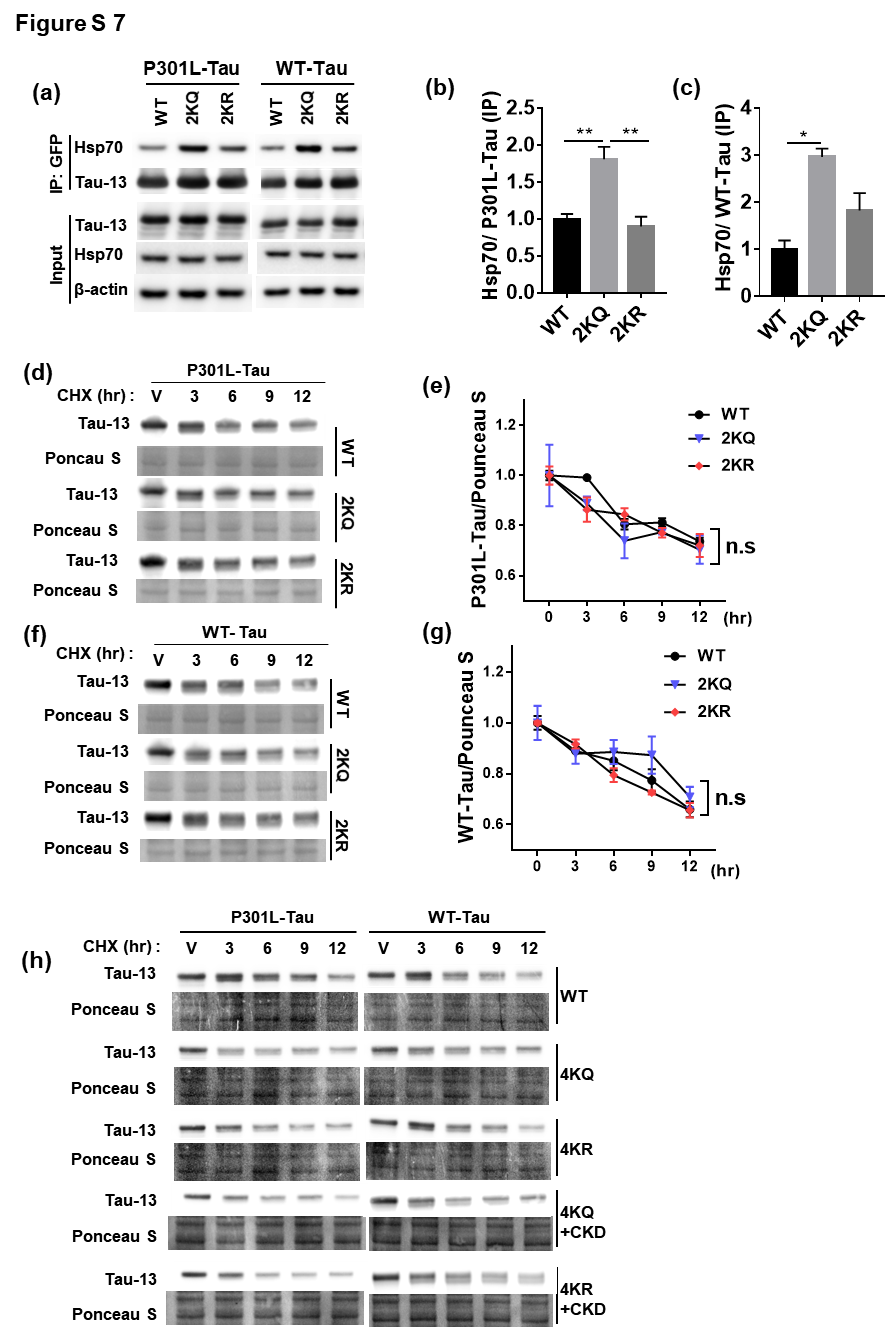


**Figure S7.** **The effects of HDAC6 regulated acetylation sites of tau on interactions with chaperones and tau, and degradation of tau**

(a-c) Acetyl-mimic and -silencing mutations on K274 and K321 of P301L and WT tau regulated interactions with chaperones in HT22 cells. Representative immunoblot images (a) and quantification (b,c) (n=3, independent experiments). Data are presented as means ± SEM. One-way ANOVA followed by Bonferroni post-hoc test. **P* < 0.05, ***P* < 0.01 (d-g) Degradation rates of tau by mutations. Representative immunoblot images (d,f) and quantification (e,g). Data are presented as means ± SEM. Analysis of covariance (ANCOVA). n.s.: non significant. (h) Representative immunoblot of altered degradation rate of tau by mutations and CKD-504. (Related to Figure 6f-k).


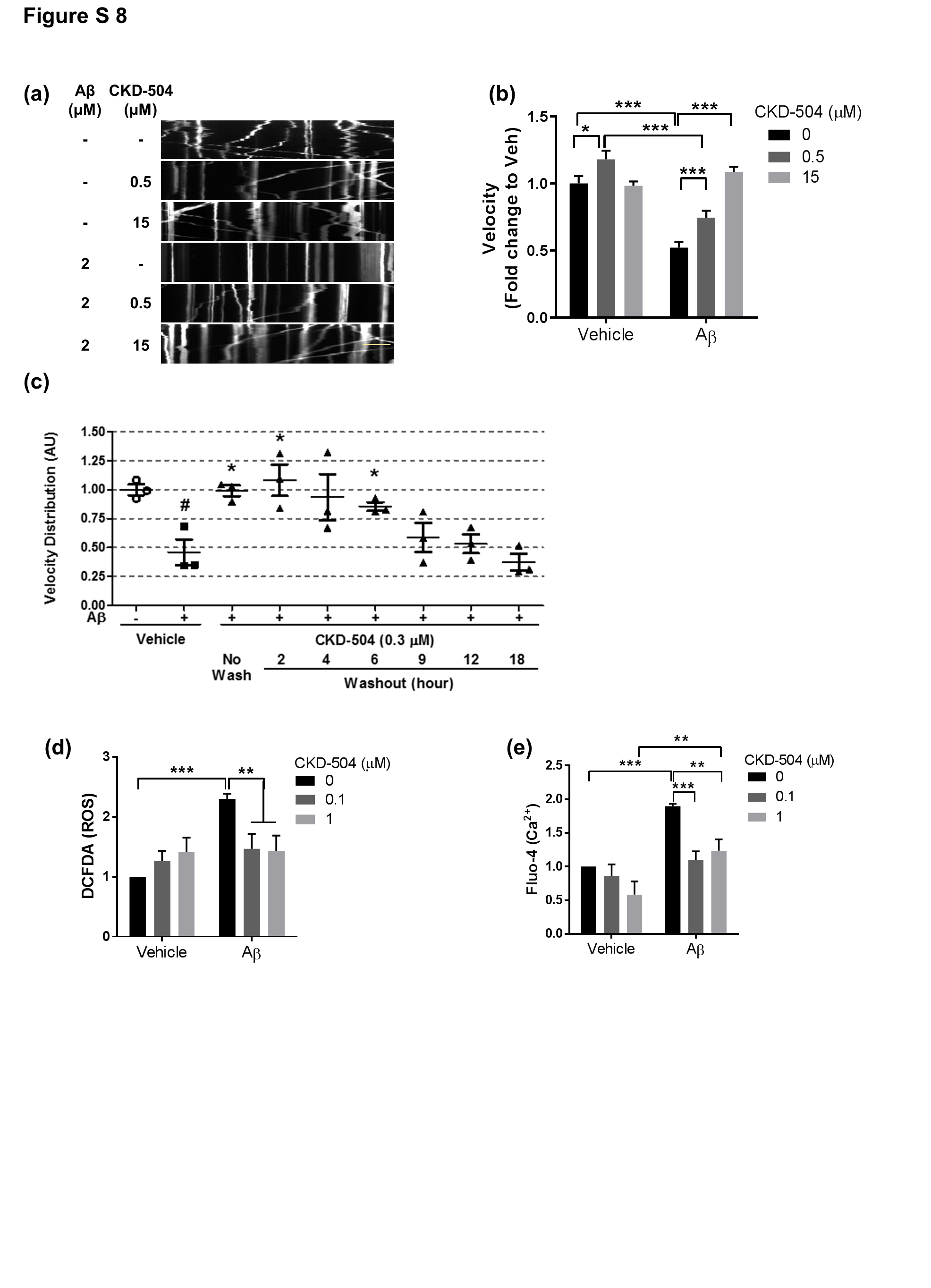


**Figure S8.** **CKD-504 rescued Aβ toxicities such as deficit of mitochondrial axonal transport, excessive ROS and Ca^2+^ *in vitro*.**

(a), (b) The deficit in mitochondrial axonal transport by Aβ (2 μM) was rescued by CKD-504 in primary rat hippocampal neurons. Representative kymographs (a) and quantification of velocity of mitochondria (b) 4 independent experiments (n=30 cells per group). (c) Prolonged effect of CKD-504 after washout. Velocity of mitochondria axonal transport was evaluated after CKD-504 washout in cultures of Aβ-treated primary rat hippocampal neurons. Data are presented as means ± SEM. Student’s *t*-test, #*P* ≤ 0.05 vs. vehicle treated group; ** P* ≤ 0.05 vs. Aβ-treated group (d) Increased ROS by Aβ (2 μM) was recovered by CKD-504 in HT22 cells, which was analyzed by DCFDA assay (n=13, independent experiments). (e) Elevated Ca^2+^ level by Aβ (2 μM) was rescued by CKD-504 in HT22 cells, which was analyzed by Fluo-4 assay (n=5, independent experiments). Scale bar: 10 μm. Data are presented as means ± SEM. Two-way ANOVA followed by Bonferroni post-hoc test. **P* < 0.05, ***P* < 0.01, ****P* < 0.01.
